# Dimerization of the 4Ig isoform of B7-H3 in tumor cells mediates enhanced proliferation and tumorigenic signaling

**DOI:** 10.1038/s42003-023-05736-8

**Published:** 2024-01-05

**Authors:** Margie N. Sutton, Sarah E. Glazer, Riccardo Muzzioli, Ping Yang, Seth T. Gammon, David Piwnica-Worms

**Affiliations:** Department of Cancer Systems Imaging, The University of Texas M. D. Anderson Cancer Center, Houston, TX 77030 USA

**Keywords:** Cell signalling, Tumour immunology

## Abstract

B7-H3 (*CD276*) has two isoforms (2Ig and 4Ig), no confirmed cognate receptor, and physiological functions that remain elusive. While differentially expressed on many solid tumors correlating with poor survival, mechanisms of how B7-H3 signals *in cis* (tumor cell) versus in trans (immune cell co-regulator) to elicit pro-tumorigenic phenotypes remain poorly defined. Herein, we characterized a tumorigenic and signaling role for tumor cell-expressed 4Ig-B7-H3, the dominant human isoform, in gynecological cancers that could be abrogated upon CRISPR/Cas9 knockout of B7-H3; tumorigenesis was rescued upon re-expression of 4Ig-B7-H3. Size exclusion chromatography revealed dimerization states for the extracellular domains of both human 4Ig- and murine 2Ig-B7-H3. mEGFP lifetimes of expressed 4Ig-B7-H3-mEGFP fusions determined by FRET-FLIM assays confirmed close-proximity interactions of 4Ig-B7-H3 and identified two distinct homo-FRET lifetime populations, consistent with monomeric and homo-dimer interactions. In live cells, bioluminescence imaging of 4Ig-B7-H3-mediated split luciferase complementation showed dimerization of 4Ig-B7-H3. To separate basal from dimer state activities in the absence of a known receptor, C-terminus (cytosolic) chemically-induced dimerization of 4Ig-B7-H3 increased tumor cell proliferation and cell activation signaling pathways (AKT, Jak/STAT, HIF1α, NF-κβ) significantly above basal expression of 4Ig-B7-H3 alone. These results revealed a new, dimerization-dependent intrinsic tumorigenic signaling role for 4Ig-B7-H3, likely acting *in cis*, and provide a therapeutically-actionable target for intervention of B7-H3-dependent tumorigenesis.

## Abstract (web summary)

The 4Ig isoform of B7-H3, an immune modulatory protein expressed in tumors, resides in dimeric states with enhanced dimerization-dependent intrinsic signaling and tumorigenic activities, providing a therapeutically-actionable target for intervention.

## Introduction

Ligand engagement-induced dimerization is a classic method by which cell surface signaling proteins communicate extracellular signals into a functional intracellular response. In fact, proteins in biological systems rarely act in isolation and the ability to form dimers and higher-order oligomers to elicit cellular responses is a common cell signaling phenomenon. It is evident, even with limited biophysical data, that different biostructures (e.g., enzymes, transcription factors, integral membrane receptors, etc.) capitalize on protein dimerization to regulate the control and execution of their functions. EGFR is a typical example by which ligand (EGF) engagement induces dimerization and a biochemical signal can be transduced, activating kinase signaling cascades. Advances in spatiotemporal characterization of protein-protein interactions (PPIs) using non-invasive bioluminescence- and fluorescence-based approaches have interrogated PPIs and their structure-function relationships at multiple scales from subcellular to whole animals in real-time in vivo^1–5^, providing novel mechanistic insights into the functional roles of induced or spontaneous protein self-assembly.

In addition to regulating intracellular signaling and biochemical processes, PPIs can also occur between cells to elicit intercellular communications and extracellular responses. The fundamental understanding of immune checkpoint protein-protein interactions between cancer cells and their cognate receptor on immune cells (T-cells) has revolutionized cancer immunotherapy and led to a new era in cancer treatment. The B7-family of immune regulatory proteins are structurally related, cell-surface proteins that play a central role in both signaling in cis (within antigen-presenting cells/tumor cells) and in trans (between tumor and immune cells). Since the groundbreaking discovery of the mechanisms of immune checkpoint mediated by interactions between B7-family members, including CTLA-4, PD-1, and PD-L1, many investigations have sought to understand the fundamental functions of all family members, identify their cognate receptor(s) and characterize the resulting tumorigenic and co-regulatory functions. One extended family member, B7-H3 (*CD276*), has garnered significant attention as its protein translation is tightly regulated by microRNAs (e.g., mir29) and overexpression of B7-H3 has been observed on the tumor cell surface and vascular endothelium for many human tumor types^6–8^. The correlation between B7-H3 protein expression and poor prognosis for a number of solid human tumors, including cervical, ovarian and breast cancers among many others, has been demonstrated extensively over the last decade^6,8–10^. In addition, recent studies have directly linked tumor-expressed B7-H3 protein with increased drug resistance^11^, promotion of metastasis^12^, and increased proliferation, invasion and migration^13,14^. This is perhaps related to its role in regulating tumor immune responses; however, challenges have remained to identify the cognate receptor(s) and thus, the role of B7-H3 in immune response, tumor immune evasion, and cancer cell-intrinsic tumorigenic signaling are uncertain^15^.

While all members of the B7 and B7H families share structural features and sequence similarity, their individual capacity to recognize and regulate other members of the CD28/B7 family remains unclear. This, paired with the complexity of their expression patterns and regulation across various immune and cancer cell types, highlights the intricacy of their functional contributions to regulation of immune responses. Through examination of the evolutionary selection of costimulatory ligands and receptors across the B7/CD28 family of immunoregulatory proteins, studies have tried to identify key features with the potential to mediate a balance between immune responsiveness and autoimmunity. Importantly, common stalk domains and transmembrane domains of several of the B7-family members have been identified by such analysis, suggesting functional significance that may modulate the capacity of ligand/receptor binding through both direct and indirect mechanisms^16–18^. Like other cell surface receptors that are responsible for communicating cell-to-cell information, B7-H3 *(CD276)* contains extracellular immunoglobulin variable (IgV) and immunoglobulin constant (IgC) domains that are likely related to cognate binding, a stalk region, a helical transmembrane domain (AA 467-487), and a cytoplasmic domain. Initially, B7-H3 was thought to co-stimulate the immune response^19^, but the predominance of recent studies have shown that it has a co-inhibitory role on human T-cells, contributing to cancer cell immune evasion^20–22^. The confounding results may be in part due to a species difference when interpreting previously generated data using murine models, as humans contain both 4Ig and 2Ig isoforms of the protein, while mice and other rodents only contain a 2Ig isoform^23,24^. Murine 2Ig-B7-H3 and human 2Ig-B7-H3 share 88.6% protein homology, and human 4Ig-B7-H3 contains a tandem repeat of the extracellular IgV and IgC domains. Interestingly, the crystal structure of the extracellular domain of murine 2Ig-B7-H3 identified a putative dimerization interface^21^ and evidence suggests that other B7 family members may also adapt a homodimeric structure related to their active signaling state^25^. However, the structural-functional state of activated B7-H3, particularly in living cells, is unknown.

Therefore, to test the hypothesis that human 4Ig-B7-H3 dimerizes in live cells, a custom-designed split-luciferase reporter system was developed that directly identified dimerization in real time in tumor cells by bioluminescence imaging, which was independently confirmed by fluorescence lifetime microscopy. Furthermore, the functional significance of 4Ig-B7-H3 dimerization/multimerization was interrogated in vitro and in vivo, providing evidence of tumor cell-intrinsic signaling and enhanced tumor growth. In addition, an inducible C-terminus (cytosolic) dimerization system was generated to distinguish functional consequences of protein expression per se from dimerization. Differential activation of tumorigenic and cell activation signaling pathways (AKT, Jak/STAT, HIF1α, and NF-κβ) increased substantially upon chemically-induced dimerization of 4Ig-B7-H3 overexpression at equilibrium of 4Ig-B7-H3 alone, providing mechanistic evidence for dimerization-mediated tumorigenic signaling. These data revealed a new, dimerization-dependent tumorigenic role for 4Ig-B7-H3 and provided a therapeutically actionable target for intervention of 4Ig-B7-H3-driven tumorigenesis.

## Results

### Knockout of B7-H3 expression reduced clonogenic growth and tumorigenic signaling

While dimerization motifs have been proposed based on the crystal structure of the extracellular domain of murine 2Ig-B7-H3^21,26^, it was unclear if this was a packing artifact that may have occurred during the crystallization process. Indeed, no direct evidence of dimerization or higher order protein-protein interactions related to function has been previously published for B7-H3. Thus, to test the hypothesis that dimerization modulates B7-H3 function, we developed several molecular and biochemical tools to investigate the contribution of protein expression of B7-H3 versus protein dimerization on intrinsic tumorigenic signaling in live tumor cells.

**Fig. 1:**
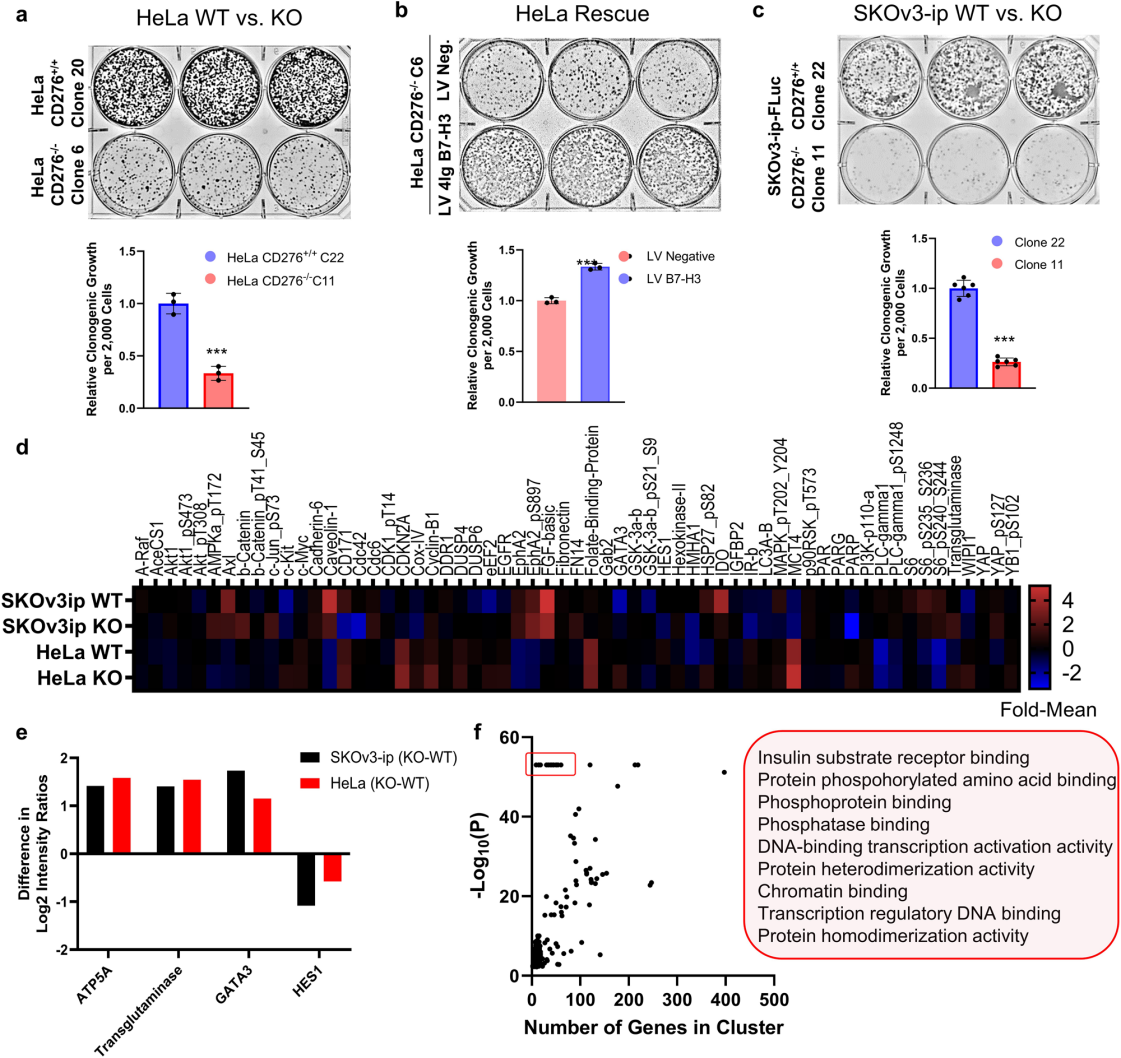
Knockout of B7-H3 reduces clonogenic growth and tumorigenic signaling in gynecological cancer cells. **a** 2000 HeLa wildtype (Clone 20) or *CD276* knockout cells (Clone 6) were seeded and allowed to grow for 10 days, changing the media every 72 h. **b** HeLa CD276^-/-^ cells (Clone 6) engineered to re-express 4Ig-B7-H3 (LV B7-H3) or a negative vector control (LV Neg) were used to perform a clonogenic assay as described above. 2,000 cells were seeded and allowed to grow for 10 days, changing the media every 72 h. **c** 2,000 SKOv3-ip-FLuc wildtype (Clone 22) or *CD276* knockout cells (Clone 11) were seeded and allowed to grow for 10 days, changing the media every 72 h. **a-c** Clonogenic growth was quantified using ImageJ and represented in the bar graph (mean ± SD). The experiment was performed in technical triplicate with three biological replicates, ***p < 0.001 as determined by Student’s two-tailed t-test. **d** Reverse phase protein lysate array analysis and heat map visualization of the median normalized expression of each protein with and without 4Ig-B7-H3 expression for SKOv3-ip and HeLa cell lines. **e** Difference (KO-WT) in normalized protein expression for a subset of concordant proteins between SKOv3-ip and HeLa cell lines. **f**. X-Y plot of -Log(*P* values) and number of genes identified in each GO Term enrichment analysis with key pathway enrichment annotated.

First, to confirm previous reports of a tumorigenic role for B7-H3 protein expression^6,27^, we used CRISPR/Cas9 to knockout *CD276* (B7-H3) expression in HeLa cervical cancer cells **(**Supplemental Fig. 1**)**. We generated two stable HeLa cell line pairs, WT (C20 and C22) and *CD276* knockout (KO) (C6 and C11), respectively, each grown from a single clone to reduce the effect of clonal-specific cell line alterations. All cell lines were validated by both immunofluorescence, documenting the expected membranous localization of 4Ig-B7-H3 **(**Supplemental Fig. 1b, c**)**, and Western blot protein expression **(**Supplemental Fig. 1d, full blot Supplemental Figure**)**. Lentiviral re-expression of 4Ig-B7-H3 or a non-targeting control vector with pooled selection was used to generate HeLa rescue cell lines for the studies **(**Supplemental Fig. 1c). Knockout of *CD276*^*-/-*^ reduced 10-day clonogenic growth relative to HeLa WT cells **(**Fig. 1a**)**. Lentiviral re-expression of 4Ig-B7-H3 on the KO background rescued the phenotype, wherein re-expression of 4Ig-B7-H3 protein resulted in increased 10-day clonogenic growth compared to lentiviral control cells **(**Fig. 1b**)**. Experiments using the second pair of HeLa WT and KO clones, with their respective lentiviral rescue pairs revealed similar results, wherein KO of B7-H3 reduced clonogenic growth while re-expression increased clonogenic growth **(**Supplemental Fig. 2a, b**)**. Additionally, we generated a second knockout model using the SKOv3-ip-FLuc cell line **(**Supplemental Fig. 1e**)**. This phenotype was also observed in two paired SKOv3-ip-FLuc CD276^+/+^ WT (Clone 10 and 22) and CD276^-/-^ KO (Clone 4 and 11) cell lines, respectively, confirming a generalizable phenotype **(**Fig. 1c and Supplemental Fig. 2c**)**. Similarly, short term cell growth was measured after 72 h for both HeLa and SKOv3-ip WT cells and paired KO cell lines by sulforhodamine B staining, wherein knockout of *CD276* resulted in decreased cell growth by 50%-80% **(**Supplemental Fig. 2d, e**)**. Using our paired WT and *CD276*^*-/-*^ knockout cell lines (HeLa and SKOv3-ip), we next compared live cell (in vitro) tumorigenic signaling between the paired cell lines. Reverse phase protein array (RPPA) analysis was used to quantify hundreds of proteins (499) in cell lysates obtained at resting state from WT or *CD276*^*-/-*^ KO cells. Using this high-throughput proteomics approach, we probed the differences in protein expression and phosphorylation for a wide array of targets known to be important in cancer cell signaling, proliferation, metastasis, and immune modulation. In *CD276*^*-/-*^ knockout cells, decreased phosphorylation of AKT pT308 and pS473, S6 pS235-S236, and decreased cMYC, IGFBP2, fibronectin, and CDKN2A expression was found, amongst others **(**Fig. 1d**)**. The difference in protein expression between KO and WT cells were compared for both SKov3-ip and HeLa cell lines and the mean difference on a per-protein basis was calculated. Concordance between *changes* in protein expression that were common between both SKOv3-ip and HeLa cells were identified **(**Supplemental Fig. 3**)**. Expression of ATP5A, transglutaminase and GATA3 were significantly increased upon knockout of B7-H3, while HES1 was significantly decreased **(**Fig. 1e**)**. Gene Ontology analysis^28^ of the mean differentially expressed or activated proteins between WT and B7-H3 KO cells between both cell lines revealed anticipated enrichment of biological processes relating to key phosphate-related cell signaling activation, including insulin substrate receptor binding, intracellular signal transduction, cell proliferation and transcriptional activation. To eliminate a potential bias introduced by the pathway size perturbed^29^, the -Log(p-Value) for each GO term was plotted against the number of genes included in the biological process **(**Fig. 1f**)** and the resulting highly significant GO terms with small gene sets (red box) were used to identify the pathways in which perturbation would be most significantly affected upon loss of B7-H3 **(**Fig. 1f**)**. The entire list of GO annotations for the mean difference in protein expression between WT and KO cells for both cell lines can be found in Supplemental Data 1. The top 10 GO annotations for the differentially expressed genes between HeLa WT and KO cells can be found in Table 1 while the entire list can be found in Supplemental Data 2. These results reinforce a growing body of literature that implicates B7-H3 in cancer cell proliferation and tumorigenic signaling where PI3K^30,31^, Src/STAT3^32^ and MEK^33^ were previously identified as functional signaling nodes in B7-H3-supported cancer cell proliferation.

**Table 1:** The top 10 most statistically significant Gene Ontology terms of differentially expressed proteins and their phosphorylation states between HeLa wildtype and B7-H3 KO cells as determined by RPPA Analysis.

OID	Term	*P*-value	Number	Annotated genes
			annotated	
GO:0006796	phosphate-containing compound metabolic process	3.45E-09	23	EEF2K, EGFR, GPL127, FN1, JUN, INSR, ARAF, COX4I1, PLCG1, Epha2, AXL, AKT1, PIK3CA, CDKN2A, TSC2, GSK3B, HK2, PRKCI, DDR1, Lysmd2, Hes1, PARG, ACSS2
GO:0006793	phosphorus metabolic process	4.11E-09	23	EEF2K, EGFR, GPL127, FN1, JUN, INSR, ARAF, COX4I1, PLCG1, Epha2, AXL, AKT1, PIK3CA, CDKN2A, TSC2, GSK3B, HK2, PRKCI, DDR1, Lysmd2, Hes1, PARG, ACSS2
GO:0007167	enzyme linked receptor protein signaling pathway	8.40E-09	15	EGFR, JUN, INSR, IGFBP2, PLCG1, EPHA2, AXL, AKT1, PIK3CA, TSC2, GSK3B, PRKCI, DDR1, HES1, GRB2
GO:0016310	phosphorylation	1.28E-08	20	EEF2K, EGFR, MYC, FN1, JUN, INSR, ARAF, COX4I1, EPHA2, AXL, AKT1, PIK3CA, CDKN2A, TSC2, GSK3B, HK2, PRKCI, DDR1, LYSMD2, HES1
GO:0007169	transmembrane receptor protein tyrosine kinase signaling pathway	2.11E-08	13	EGFR, INSR, IGFBP2, PLCG1, EPHA2, AXL, AKT1, PIK3CA, TSC2, GSK3B, PRKCI, DDR1, GRB2
GO:0006468	protein phosphorylation	6.01E-08	18	EEF2K, EGFR, MYC, FN1, JUN, INSR, ARAF, EPHA2, AXL, AKT1, PIK3CA, CDKN2A, TSC2, GSK3B, PRKCI, DDR1, LYSMD2, HES1
GO:0035239	tube morphogenesis	1.47E-07	13	EGFR, MYC, FN1, JUN, PLCG1, EPHA2, AKT1, PIK3CA, TSC2, HK2, PRKCI, DDR1, HES1
GO:0035556	intracellular signal transduction	4.28E-07	20	EGFR, MYC, FN1, JUN, INSR, ARAF, PLCG1, EPHA2, AXL, AKT1, PIK3CA, CDKN2A, TSC2, GSK3B, PRKCI, PRS6, LYSMD2, HES1, ARHGAP45, GRB2
GO:0009653	anatomical structure morphogenesis	9.02E-07	19	EEF2K, EGFR, MYC, FN1, JUN, INSR, PLCG1, EPHA2, AXL, AKT1, PIK3CA, TSC2, GSK3B, HK2, PRKCI, PRS6, DDR1, HES1, GRB2
GO:0001932	regulation of protein phosphorylation	9.60E-07	15	EEF2K, EGFR, MYC, FN1, JUN, INSR, ARAF, EPHA2, AKT1, PIK3CA, CDKN2A, TSC2, PRKCI, LYSMD2, HES1

### Tumor burden depends on B7-H3 expression

**Fig. 2:**
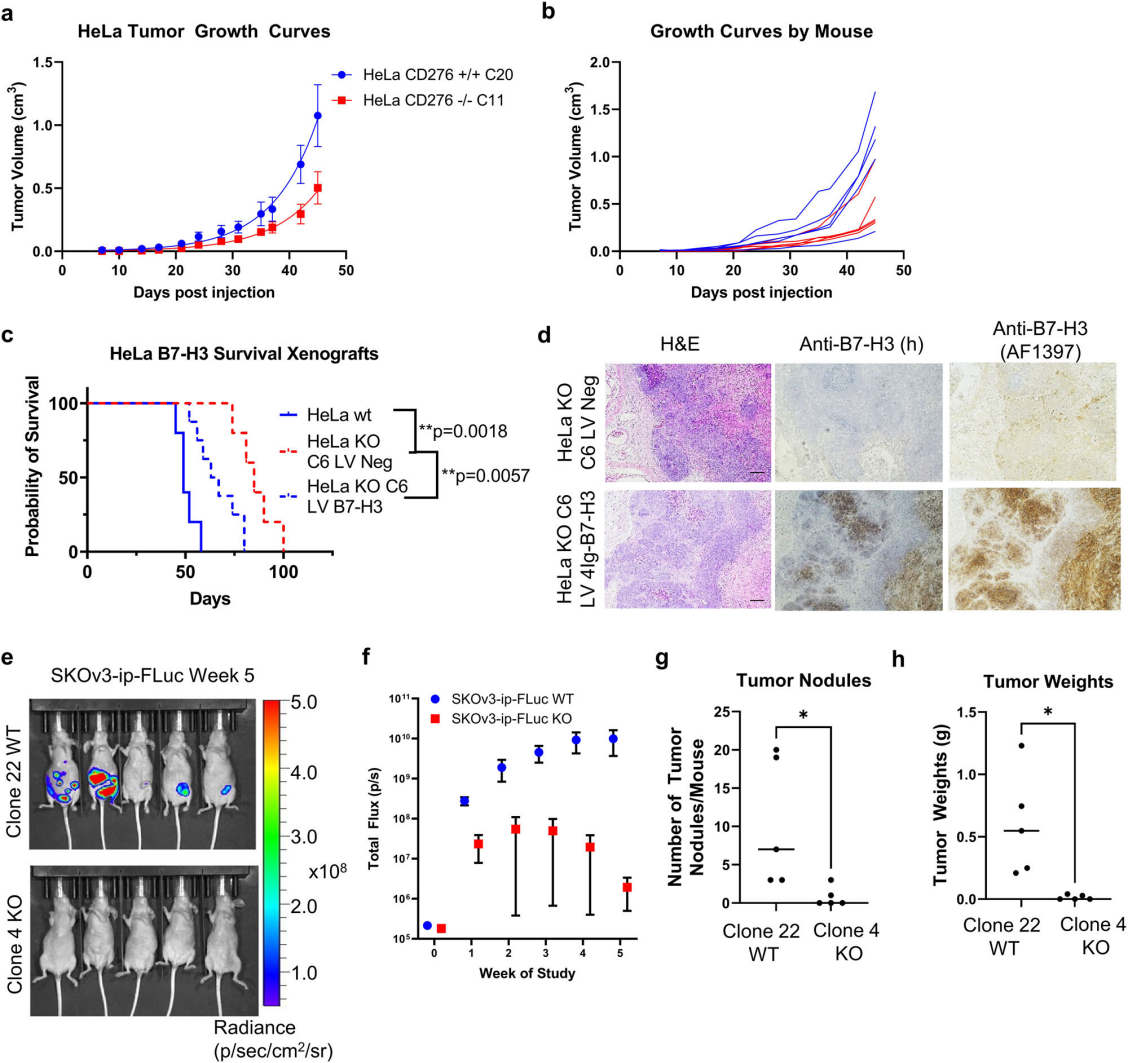
Tumor burden depends upon expression of 4Ig-B7-H3. **a**–**d** HeLa WT B7-H3, KO or rescue xenografts were implanted on the right flank of *nu/nu* mice. Tumor growth was monitored and plotted as aggregates (**a**) or independently by mouse (**b**) and endpoint survival was documented in a Kaplan-Meier plot **c**. Statistically significant difference in survival between HeLa *CD276*^+/+^ (*n* = 5) and HeLa *CD276*^-/-^ (*n* = 9) was determined (*p* < 0.0018, Mantel-Cox Log-rank test). HeLa KO cells were used to re-express 4Ig-B7-H3 using a lentiviral vector versus negative control. Rescue expression of 4Ig-B7-H3 in tumor xenografts was measured. Lentiviral re-expression of 4Ig-B7-H3 (HeLa KO C6 LV 4Ig-B7-H3; n = 10) reduced the median survival (*p* = 0.0057) compared to KO xenografts (HeLa KO C6 LV Neg; *n* = 9) (*p* < 0.0057, Mantel-Cox Log-rank test). **d** Endpoint histology was performed and representative H&E staining and human 4Ig-B7-H3 immunostaining is shown. Images captured with 4X objective, scale bars, 50 µm. **e**–**h**. SKOv3-ip-FLuc orthotopic tumors were injected intraperitoneally into *nu/nu* mice. Tumor growth was monitored by firefly luciferase bioluminescence **e** and plotted as aggregates (Mean ± SEM) **f**. Endpoint tumors (week 5) were collected and nodule numbers **g** and tumor weight **h** were measured. KO of B7-H3 tumor expression resulted in decreased tumor burden as determined by bioluminescence (*p* = 0.0385), number of nodules (p < 0.001) and tumor weight (p < 0.001).

To further interrogate the consequences of 4Ig-B7-H3 loss, we compared tumor burden and overall survival of mice harboring WT or B7-H3 deficient tumors in vivo. Subcutaneous xenografts of HeLa WT and KO tumors were measured bi-weekly and tumor burden as well as overall survival assessed. Knockout of B7-H3 significantly reduced tumor growth rates **(**Fig. 2a-b**)** and extended overall survival of animals compared to those implanted with WT (B7-H3 expressing) tumors (*p* = 0.0018) **(**Fig. 2c**)**. Similar to that observed in vitro, lentiviral re-expression of B7-H3 using a paired cell line (C6), rescued the tumorigenic phenotype, reducing the median survival from 85 days (KO LV Neg) to 65 days (rescued LV B7-H3) (*p* = 0.0057) **(**Fig. 2c**)**. Immunohistochemistry of endpoint tumors documented B7-H3 expression in rescued tumors as expected **(**Fig. 2d**)**. Similarly for SKOv3-ip-FLuc paired WT and KO orthotopic tumors, wherein tumor burden was non-invasively monitored using firefly luciferase as a surrogate for tumor cell growth and metabolism, reduced growth rates and tumor burden were observed with B7-H3 KO tumors **(**Fig. 2e-f**)**. Endpoint tumor weights **(**Fig. 2g**)** and nodules **(**Fig. 2h**)** were counted at 5 weeks post implantation, where WT tumor burden was significantly higher compared to KO tumors. Thus, these studies confirmed previous reports of the tumor-intrinsic proliferative role of B7-H3 expression and provided rationale for our studies to further investigate the structural-functional state of B7-H3 as related to this enhanced tumorigenic function.

### Prediction of B7-H3 dimerization by molecular modeling and analytical SEC of the extracellular domain of 4Ig- and 2Ig-B7-H3 in vitro

**Fig. 3:**
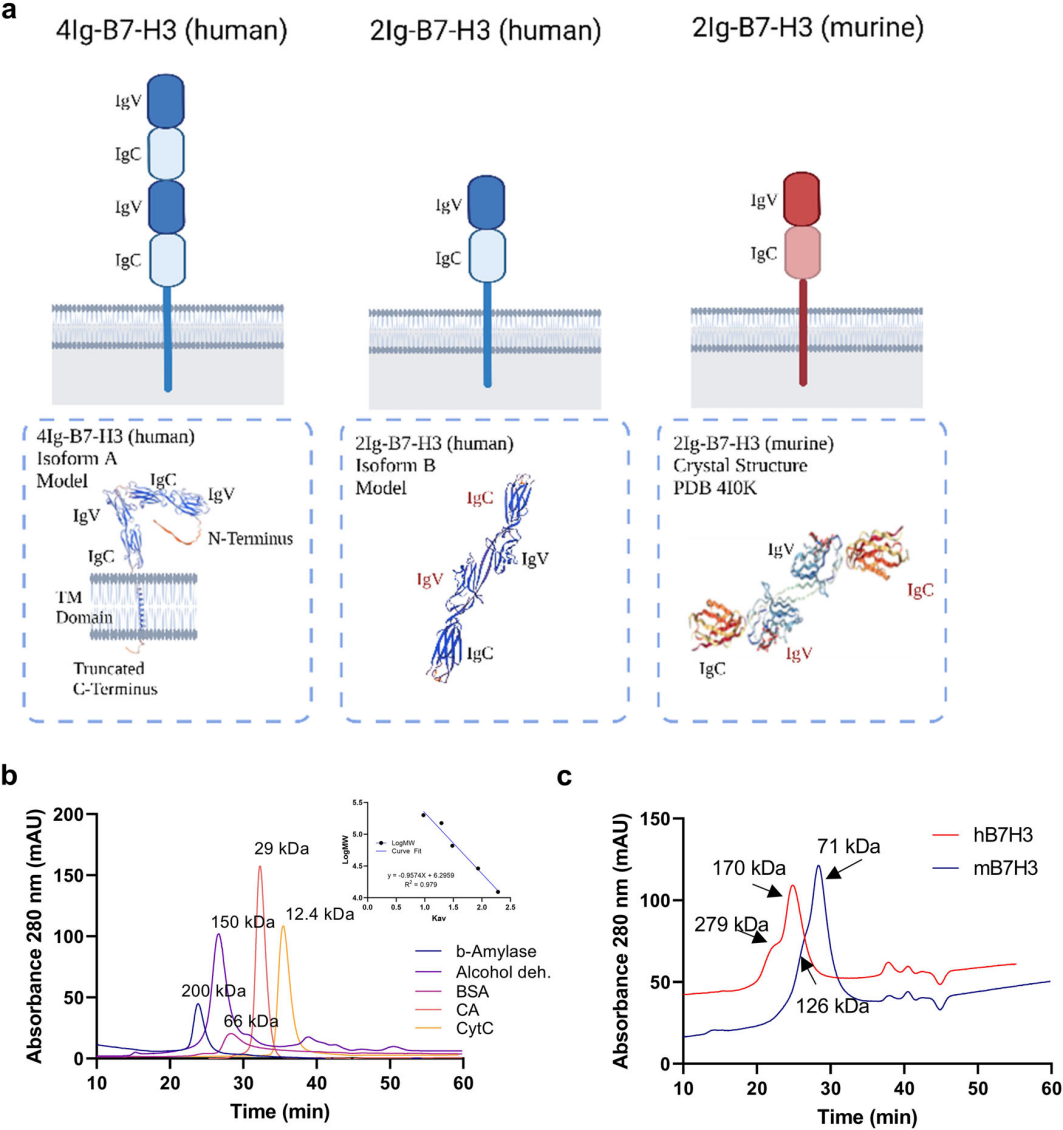
B7-H3 isoforms share homology across species and the extracellular domain may confer a dimeric state. **a** Model depictions of 4Ig-B7-H3 and 2Ig-B7-H3 human and murine isoforms with their predicted (human) or known crystal structure (murine). B7-H3 is a type I transmembrane protein which contains an extracellular, a transmembrane and a short intracellular domain. Human B7-H3 exists in two isoforms as determined by its extracellular domain: 4Ig-B7-H3 which comprises two identical pairs of immunoglobulin variable (IgV)-like and immunoglobulin constant (IgC)-like domains, and 2Ig-B7-H3 which comprises a single pair of IgV-like and IgC-like domains. Murine B7-H3 exists only as the 2Ig-B7-H3 isoform. IgV: immunoglobulin variable; IgC: immunoglobulin constant. **b** Size exclusion chromatography (SEC) of molecular weight standards and the associated calibration curve (inset). **c** Chromatograms of human 4Ig-B7-H3 and murine 2Ig-B7-H3 with their calculated molecular weights.

Given that the crystal structure of the extracellular domain of murine 2Ig-B7-H3 identified a potential dimerization domain^21^, we sought to determine the potential for the human 4Ig- and 2Ig-B7-H3 isoforms to dimerize. SWISS-MODEL was used to perform protein structure homology modelling for the human protein, based on the alignment of target sequence and template structure of the murine 2Ig-B7-H3 deposited crystal structure (PDB 410 K). The resulting tertiary models predicted two distinct patterns of potential dimerization, where the human 2Ig-B7-H3 isoform closely resembled the murine 2Ig-B7-H3 crystal structure; however, the human extracellular domain of 4Ig-B7-H3 modeled as a monomer with a distinct fold between the tandem repeats **(**Fig. 3a**)**, providing an alternative surface for a dimerization interface. To determine whether the extracellular domains of murine 2Ig- and human 4Ig-B7-H3 protein had the potential to dimerize in vitro, functionally active recombinant protein (R&D Systems) was used for size exclusion chromatography (SEC) to assess the aggregation state (dimer or multimer) in solution by evaluating the apparent experimental molecular weight^34,35^. Using a SuperDex 200 10/300 column with PBS + 0.1 M NaCl + 0.05% NaN3 as eluent (flow 0.5 ml/min), a standard curve was created using proteins of known molecular mass at 0.5 mg/ml concentration and their elution times recorded **(**Supplemental Table 1**)**. Next, human 4Ig-B7-H3 (amino acids: Gly27 – Thr461) (100 μg/mL) and murine 2Ig-B7-H3 (amino acids: Val29 – Phe244) (100 μg/mL) were evaluated under the same conditions, elution times acquired, and the experimental molecular weights derived by plotting the logarithm of the molecular weight (LogMW) against the partition coefficients (K_av_), which were defined by V_e_-V_0_/V_t_-V_0_, where V_e_ represented the elution volume, V_0_ represented the void volume, and V_t_ represented the total volume **(**Fig. 3b**)**. Based on the elution times measured at the maximum peaks and the equation derived from the molecular weight calibration curve, we estimated two potential active species of human 4Ig-B7-H3 at ~279 kDa and ~170 kDa, and of murine 2Ig-B7-H3 at ~126 kDa and ~71 kDa (Fig. 3c**)**. Experimental molecular weights are highly dependent upon the form of the protein system under examination, where the shape of the protein, dimerization or multimerization status, and the interaction with the solvent can cause discrepancies between the calculated molecular weight and the experimental apparent molecular weight, a well-established principle of protein biochemistry^35,36^. As given, these data suggested that the extracellular domains of both murine 2Ig and human 4Ig isoforms of B7-H3 exist as homodimers, wherein the apparent molecular mass corresponding to the peak protein elution at 25.0 and 28.5 min, respectively, approached the sum of two molecules of the monomeric form for each species. Thus, these biochemical data led us to further interrogate the potential of B7-H3 homodimerization and potential functional consequences of this PPI in cells.

### 4Ig-B7-H3 forms homodimers/multimers in cells

**Fig. 4:**
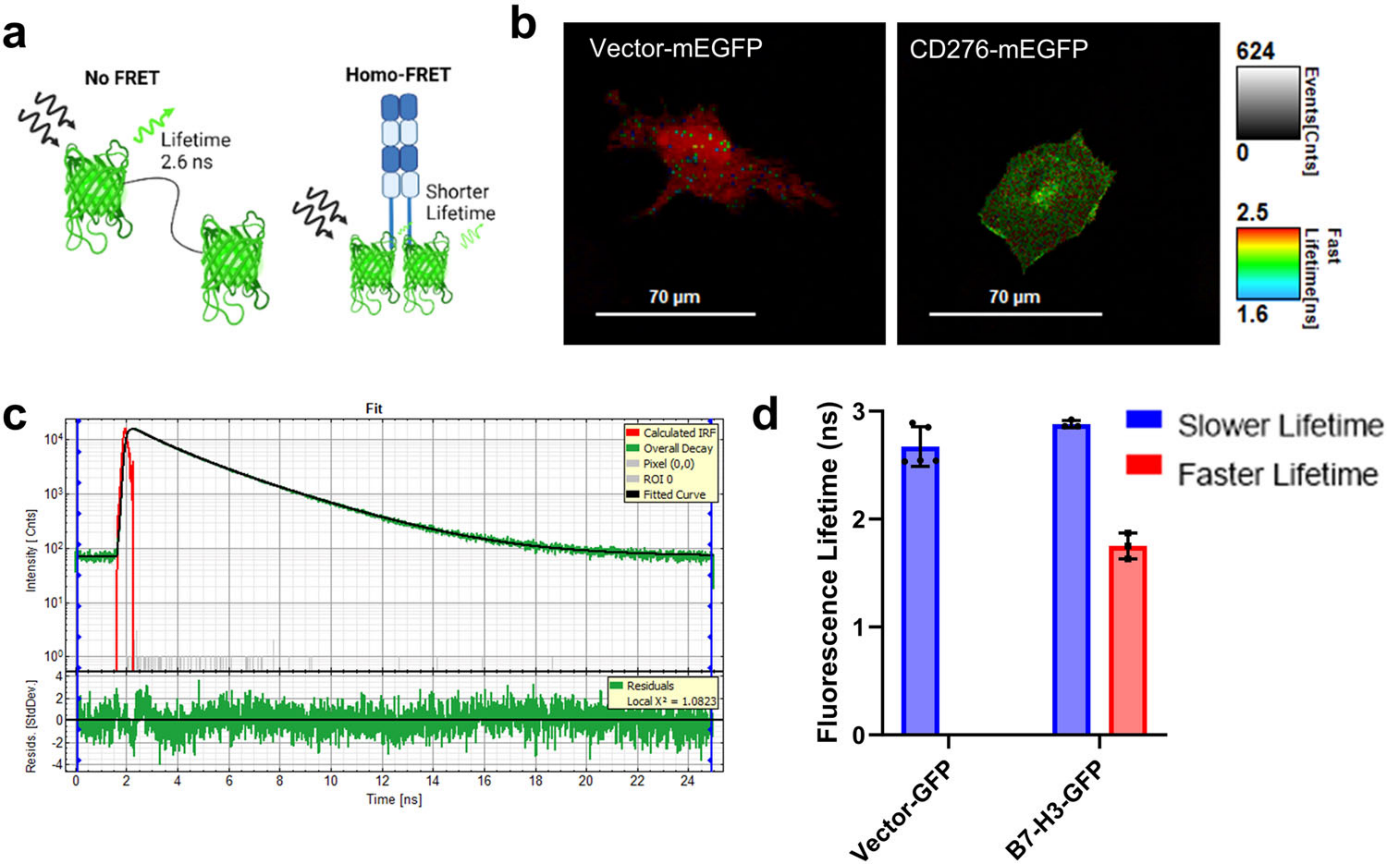
4Ig-B7-H3 dimerization detected in fixed cells using FRET-FLIM. **a** The scheme for fluorescence lifetime changes observed in a monomeric versus dimeric example, where monomeric GFP lifetime is measured at 2.6 ns compared to GFP lifetime measurements under homo-FRET conditions that results in a reduced lifetime as energy transfer occurs between the two fluorescent proteins when their localization is less than 10 nm apart. **b** Representative images showing fluorescence lifetime of monomeric GFP exogenously expressed in an empty vector, or C-terminally linked to *CD276* (4Ig-B7-H3). **c** A representative curve-fit for the lifetime decay of CD276-mEGFP using a bi-exponential fit. Calculated IRF (red), Overall decay (green), Fitted Curve (black), Residual pixels (green) are plotted below. **d** The average lifetime of GFP fluorescence determined for individual HeLa *CD276*^*-/-*^ KO cells transiently transfected with *Vector-mEGFP* or *CD276-mEGFP*, where a mono-exponential fit was the best fit for Vector-mEGFP (average lifetime 2.62 ± 0.1141 ns) and a bi-exponential fit was the best fit for CD276-mEGFP (2.57 ± 0.1658 ns and 1.33 ± 0.0939 ns). Bars represent the mean (± standard deviation) lifetimes measured for 10 individual cells over three biological replicates.

Given the complexity of live cell membranes, in vitro multimerization of the extracellular domain does not guarantee multimerization of the full protein in a living membrane. Thus, two independent methodologies were employed to test the status of 4Ig-B7-H3 dimerization in membranes. First, homo-FRET fluorescence lifetime imaging microscopy (FRET-FLIM) was used to determine whether 4Ig-B7-H3 dimerized/multimerized. This technique allowed us to capitalize on its advantages over ratiometric fluorescence or other intensity-based measurements of fluorescence resonance energy transfer (FRET). FRET-FLIM measurements are independent of local fluorophore concentration or wavelength-dependent light scattering and can deconvolute both FRET and non-FRET components, allowing measurement of the binding fraction of the FRET populations, and more specifically, allowing interrogation of close proximity (4Ig-B7-H3 dimerization) on the nanometer scale *in cellulo*^37^. If the donor and acceptor fluorophores are less than 10 nm apart, the donor fluorophore lifetime shortens proportionally to the distance between the fluorophores, allowing us to determine intra- and inter-molecular interactions. Homo-FRET-FLIM was performed by measuring the fluorescence lifetimes of human 4Ig-B7-H3 fluorescently-tagged constructs expressed on the HeLa *CD276*^*-/-*^ KO background, wherein 4Ig-B7-H3 only was expressed on a null background. Using monomeric enhanced green fluorescent protein (mEGFP), which provided a bright, photostable fluorescent protein tag and is known to have a mono-exponential decay, homo-FRET and fluorescence decays of mEGFP-tagged 4Ig-B7-H3 in fixed cells was measured by means of ultra-sensitive time-correlated single-photon counting (TCSPC) **(**Fig. 4a**)**. FRET-FLIM images also were captured for mEGFP-tagged empty Vector construct and WT 4Ig-B7-H3 cells, wherein the pseudocolor represented the EGFP lifetime in nanoseconds **(**Fig. 4b**)**. At least 10 cells per condition were analyzed and curve fitting of the decay lifetimes referenced to an instrument response function (IRF) obtained at the beginning and end of imaging using Convallaria samples was performed **(**Supplemental Fig. 4**)**. When curve fitting was applied, mono-exponential decay curves were consistently the best fit for mEGFP-tagged empty-Vector-expressing cells **(**Supplemental Fig. 5a**)**, while bi-exponential fits were more representative of the mEGFP-tagged 4Ig-B7-H3-expressing cells **(**Fig. 4c**)**. We found that the average measured lifetime for mEGFP-Vector cells was 2.62 ± 0.1141 ns, while the bi-exponential fit of mEGFP-4Ig-B7H3 cells contained two fractions, 2.57 ± 0.1658 ns and 1.33 ± 0.0939 ns, respectively **(**Fig. 4d, Supplemental Fig. 5b**)**, consistent with a mixed population of monomeric and dimeric protein complexes in resting fixed cells.

**Fig. 5:**
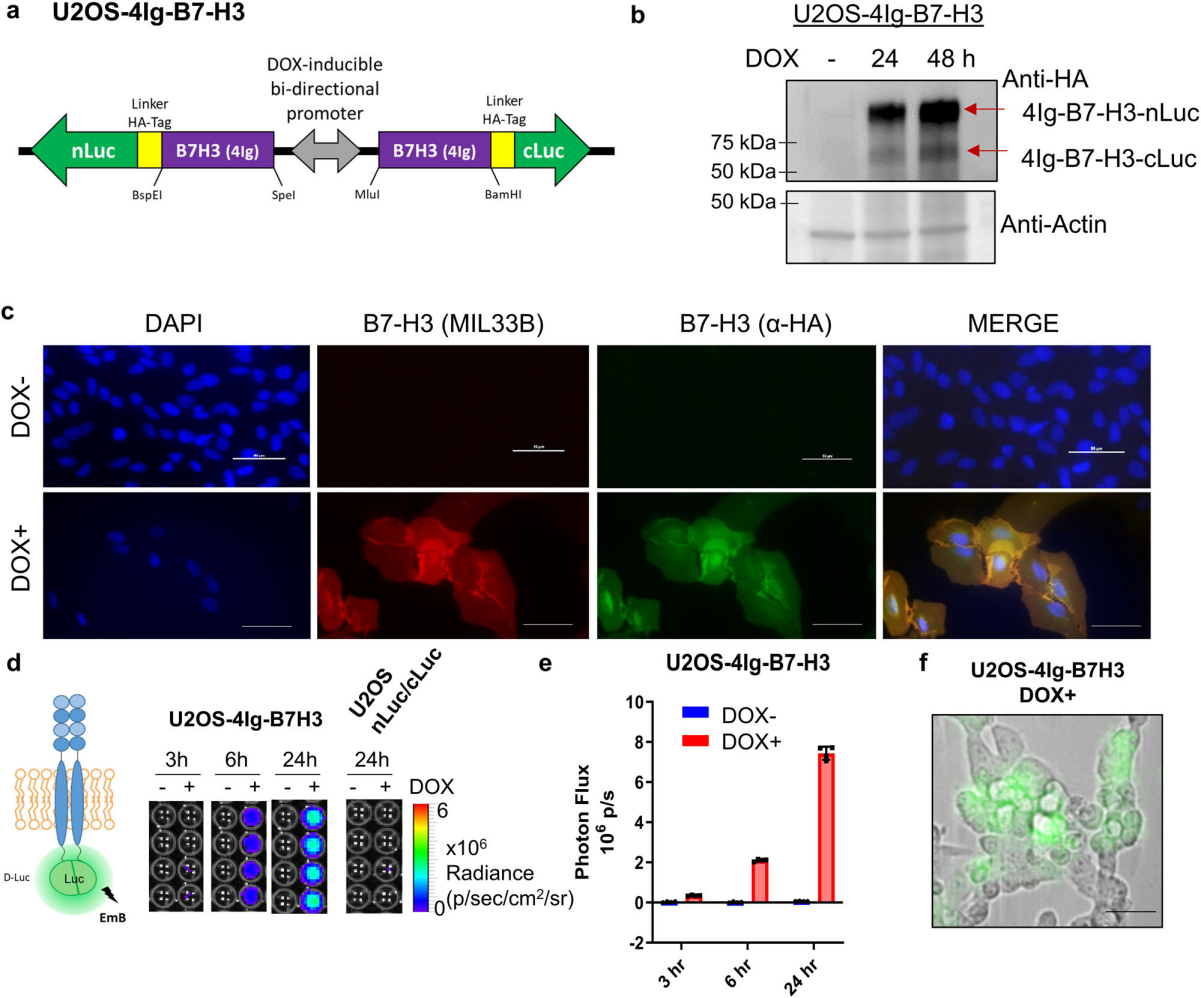
4Ig-B7-H3 forms dimers/multimers in live cells at endogenously expressed levels. **a** Expression of 4Ig-B7-H3 in the ReBiL 2.0 system is controlled by both TetR-KRAB in the absence of doxycycline (expression repression) and rtTA2^S^-M2 in the presence of doxycycline (expression induction). A bidirectional tetracycline response element (TRE_bi_) promoter controls expression of the two transgenes (human 4Ig-CD276-cLuc/nLuc fusions) simultaneously by tetracycline or doxycycline. Split-luciferase genes are linked to the 4Ig-B7-H3 proteins using a linker containing an HA-tag. **b** U2OS-4Ig-B7-H3 cells were treated with and without doxycycline for 0-48 h. Protein lysate was collected, and Western blot performed to confirm expression levels of 4Ig-B7-H3-nLuc and 4Ig-B7-H3-cLuc. Β-actin was used a loading control. **c** Confocal microscopy was performed on U2OS-4IgB7-H3 cells immunofluorescently stained with DAPI (nucleus), anti-B7-H3 (endogenous and exogenous B7-H3) and anti-HA (exogenous B7-H3) at 48 h post doxycycline induction. Expected cell membrane localization of B7-H3 was observed. Scale bars, 30 µm. **d** Model depicting 4Ig-B7-H3 homodimerization and bioluminescence complementation used throughout the study. U2OS-4IgB7-H3 cells were seeded in 96-well black-walled plates and incubated with or without doxycycline for 0-24 h. Cells were washed and incubated with fresh media and 200 nM D-luciferin for 30 min. Bioluminescent signals were measured with or without doxycycline addition (3-24 h) using an IVIS100 imaging system. Bioluminescence signal in doxycycline-induced U2OS-4Ig-B7-H3 ReBiL cells was significantly higher than in uninduced cells or empty split-Luc cells demonstrating 4Ig-B7-H3 dimerization/multimerization. **e** Photon flux was calculated over three independent experiments performed in technical triplicate and represented in the bar graph (mean ± SD). Photon flux was significantly higher (*p* < 0.001) when expression of 4Ig-B7-H3 was induced using doxycycline. **f** Bioluminescence microscopy of U2OS-4Ig-B7-H3 live cells treated with doxycycline for 24 h. BLI: 20-minute acquisition, open filter, D-Luciferase final concentration of 10 nm. 10X magnification. Scale bars, 50 µm.

To independently validate the *in cellulo* findings, we used an orthogonal second-generation recombinase-enhanced bimolecular luciferase complementation platform (ReBiL 2.0)^2,38,39^ to dynamically characterize proximity states (homo-dimerization) of 4Ig-B7-H3 in live cells. To achieve this, U2OS-ReBiL parental cells, in which the Cre-recombinase cassette was inserted into a single locus and combined with the Tet-On system, were transfected with paired 4Ig-B7-H3 split luciferase homo-dimerization constructs comprising 4Ig-B7-H3 fused to either N-Luciferase or C-Luciferase fragments and a HA tag linker **(**Fig. 5a), In addition, a bi-directional doxycycline-regulated promoter allowed expression of constructs in a highly-tunable system to study protein-protein interactions of 4Ig-B7-H3 at near physiologic levels **(**Fig. 5a and Supplemental Fig. 6, Supplemental Fig. 7c**)**. Note that endogenous B7-H3 in U2OS cells was below the limit of detection by Western blot analysis **(**Fig. 5b, Supplemental Fig. 7d**)**. Thus, upon doxycycline induction, the low background allowed examination of both the effect of protein expression as well as proximity signals (dimerization) in this system. Confocal microscopy confirmed the expected membrane localization of doxycycline-induced expression of 4Ig-B7-H3-split luciferase fusion proteins by immunofluorescence staining using either an anti-B7-H3 antibody (red) or anti-HA antibody (green) **(**Fig. 5c**)**. Next, bioluminescence was measured in live cells, and the relative photon flux was quantitatively determined with and without doxycycline-induced expression of 4Ig-B7-H3 fusions from 3 h to 48 h. Doxycycline-induced bioluminescence signals from split luciferase complementation were readily observed for U2OS 4Ig-B7-H3-nLuc/cLuc fusion cell lines, but not U2OS-nLuc/cLuc control cell lines, indicating the dynamic close proximity of tagged 4Ig-B7-H3 in living cells, consistent with homo-dimers/multimers (from now on referred to as homodimers as this is the smallest possible PPI) **(**Fig. 5d-e**)**. Signal increased with time of exposure to doxycycline consistent with additional transcription of 4Ig-B7-H3 fusions. Single-cell resolution was obtained by overlaying bioluminescence microscopy images on brightfield images (20X), confirming the cellular localization of the homo-dimerization signals **(**Fig. 5f**)**.

### 4Ig-B7-H3 dimer formation coincides with increased proliferation and increased activation of cell signaling pathways

**Fig. 6:**
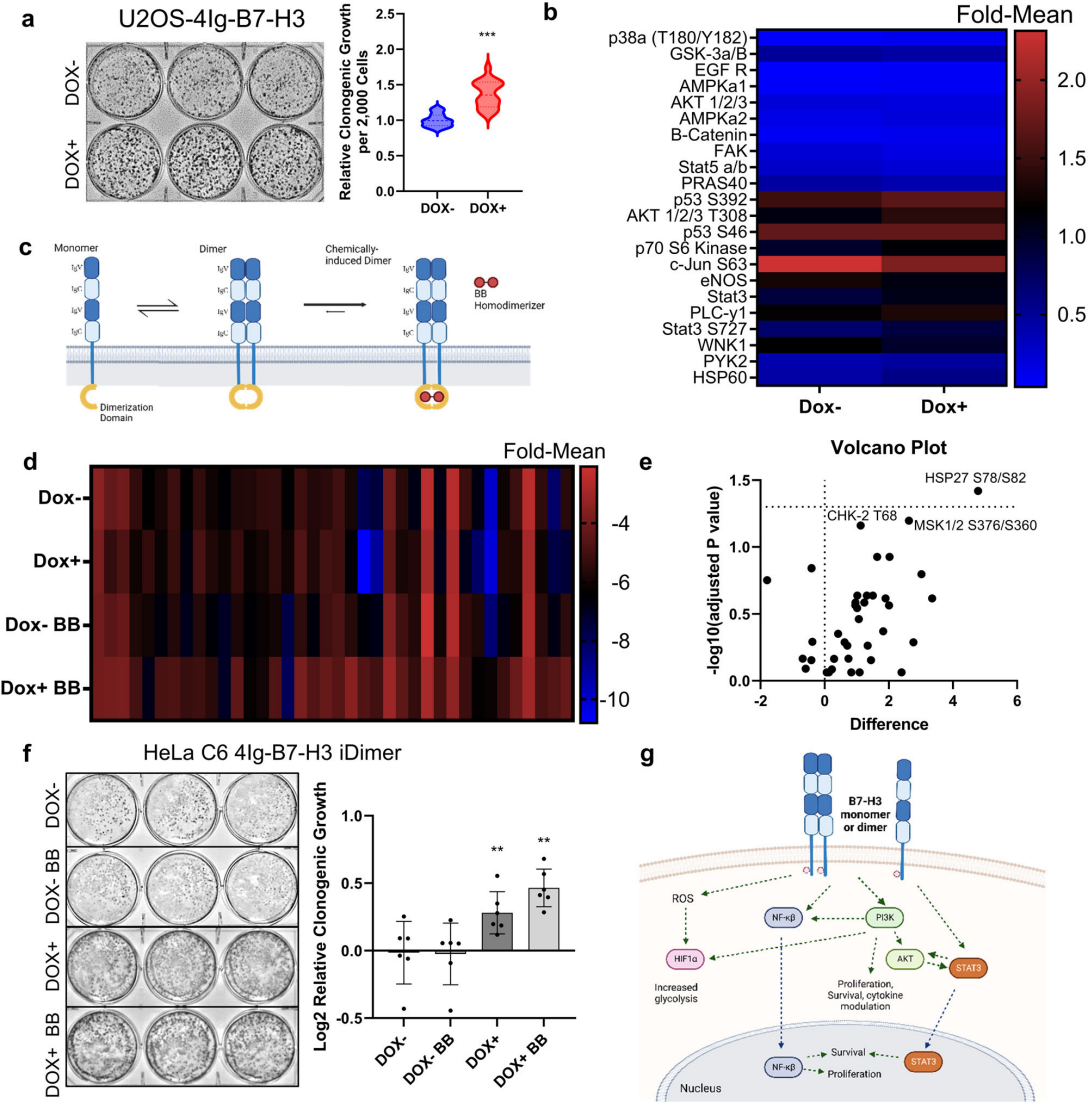
4Ig-B7-H3 dimerization drives increased proliferation and activation of tumorigenic signaling pathways. **a** 5000 U2OS-4Ig-B7-H3 cells were seeded and treated with or without doxycycline (500 µg/mL) every 3 days for two weeks. Clonogenic growth was quantified using ImageJ and represented in the bar graph (mean ± SD). The experiment was performed in technical triplicate with three biological replicates, ***p < 0.001. **b** Dox+ and Dox- U2OS-4Ig-B7-H3 cell lysates were used to perform a phospho-kinase array. Densitometry was performed using ImageJ and relative kinase activation was measured for two independent experiments. The heatmap represents the kinase signals with a greater than 2-fold change as measured between the groups. **c** Diagram representative of the doxycycline-induced 4Ig-B7-H3 expression and chemically induced dimerizer system used for sub-panels D-F. **d**. 4Ig-B7-H3-DD HeLa cells were seeded at 0.1 ×10^6^ cells/well and treated with or without doxycycline (1 ng/mL) for 48 h, and thereafter treated with BB homo-dimerizer (10 µM) for 30 min. Cell lysates were collected at each state for phospho-kinase array analysis. Densitometry was performed using ImageJ and relative kinase activation was measured for two independent experiments. Log-transformed intensities were measured for two replicate spots on the array and the mean intensity was plotted for each treatment using a heatmap representation of the data. **e** Volcano plot of kinase array data obtained as previously described in sub-panel D. Log 10 ratios between BB-treatment (DOX + , BB + ) and doxycycline-induced 4Ig-B7-H3 expression (DOX + ). Each dot represents a kinase array substrate, and those above the horizontal dotted line indicate significantly altered levels of phosphorylation (*P* < 0.05). Dots to the left of the midline of the x-axis represent substrates with decreased phosphorylation, when dimerization is induced compared to expression of 4Ig-B7-H3 alone, and those to the right are substrates with increased phosphorylation with induced dimerization. **f** 2000 HeLa 4Ig-B7-H3-DD cells were seeded and treated with or without doxycycline (1 ng/mL) for 48 h followed by the addition of 10 µM BB homo-dimerizer. Cells were allowed to grow in a clonogenic fashion for 10 days with a replacement of media and contents (doxycycline or BB) after 4 days. Clonogenic growth was quantified using ImageJ and represented in the bar graph (mean ± SD). The experiment was performed in technical triplicate with three biological replicates, **p < 0.01. **g** Diagram representative of the tumor intrinsic signaling pathways altered by 4Ig-B7-H3 expression and multimerization.

Using the U2OS-4Ig-B7-H3 split-luciferase complementation cell line, we measured clonogenic growth following doxycycline-induction at the same near-endogenous levels that 4Ig-B7-H3 dimerization/multimerization was previously identified **(**Fig. 5**)**. Long term clonogenic growth was enhanced 1.5-fold following 4Ig-B7-H3 expression and dimerization **(**Fig. 6a**)** as was short term proliferation (Supplemental Fig. 6b–d**)**. Additionally, we performed human-phosphokinase array analysis on these cell lysates following 48 h of doxycycline (0.1 μM) exposure under the identical conditions of 4Ig-B7-H3 homo-dimerization. Comparing doxycycline treated (DOX + ) to untreated cells (DOX-), we observed significant increases in several signaling pathways known to impact cancer cell proliferation and survival, including increased phosphorylation of AKT1/2/3 at T308, STAT3 S727, and p70 S6 kinase, to name a few **(**Fig. 6b**)**. Thus, 4Ig-B7-H3 dimerization coincided with increased clonogenic growth and activation of AKT signaling, the Jak/Stat pathway and modulators of HIF1α and NF-κβ pathways.

### Chemically-induced dimerization of 4Ig-B7-H3 further enhances acute signaling activation and long-term clonogenic growth

The coincidence between 4Ig-B7-H3 dimer formation and activation of tumorigenic signaling suggested the possibility that 4Ig-B7-H3 dimers directly enhanced cell activation and pro-proliferative functions; however, an alternative explanation was that the phenotype could simply result as a consequence of increased expression and membrane localization of 4Ig-B7-H3. While the construction of dimerization domain mutants might be one strategy to address this issue, mutant proteins may also result in unnatural processing/maturation and/or mis-localization of the protein. Instead, an acute chemically-inducible gain-of-function dimerization system for 4Ig-B7-H3 was developed to distinguish expression per se from dimerization, and provide direct mechanistic evidence of dimerization-mediated tumorigenic signaling. A stable HeLa cell line was generated wherein 4Ig-B7-H3 contained a C-terminal (cytosolic) FKBP-derived chemically-induced dimerization domain (DD, ~11 kDa) (hereafter referred to as 4Ig-B7-H3-DD). Gene expression was controlled using a CMV-Tet-On promoter, allowing for initiation of gene expression and basal protein homodimerization under equilibrium conditions, separately from chemically inducing a shift in state favoring full dimerization. For chemically-induced dimerization, cells were treated with a cell-permeable small molecule dimerizer, AP20187 (essentially a bis-FK506, BB homo-dimerizer), which can bind two and only two DDs^40^, therefore constraining 4Ig-B7-H3-DD into thermodynamically favored dimer states **(**Fig. 6c**)**. Using this system, signaling pathways before and after 48 h of doxycycline-induced expression of 4Ig-B7-H3-DD (basal), and after 30 min of AP20187-enhanced dimerization, were interrogated by collecting cell lysates for subsequent kinase array analysis. Log-transformed intensities were measured for two replicate spots on the array and the mean intensity was plotted for each treatment using a heatmap representation of the data **(**Fig. 6d and Supplemental Table 2**)**.

We observed the same activations of AKT signaling, the Jak/STAT pathway and modulators of HIF1α and NF-κβ by doxycycline-induced expression of 4Ig-B7-H3-DD as above, which were further increased significantly upon chemically-induced dimerization of 4Ig-B7-H3, providing direct evidence of tumorigenic signaling by 4Ig-B7-H3 dimerization. We further analyzed these data by performing multiple t-tests and utilizing a volcano plot to identify the most altered protein phosphorylation species following chemically-enhanced dimerization, i.e., those pathways significantly increased beyond expression and basal dimerization observed at equilibrium **(**Fig. 6e**)**. Upon 1 ng/mL DOX+ treatment for 48 h and 0.5 µL/mL BB treatment for 30 min, HSP27 S78/S82 phosphorylation increased significantly (p < 0.001). HSP27 is a molecular chaperone with an ability to interact with key components of the apoptotic signaling pathway, rendering an anti-apoptotic function^41^. MSK1/2 and Chk-2 T68 phosphorylation also increased beyond what was seen with expression of 4Ig-B7-H3 alone. Mitogen- and stress-activated kinases (MSK) 1 and 2 are nuclear proteins activated downstream of the ERK1/2 or p38 MAPK pathways^42^. Chk-2 phosphorylation is most notably seen as a response to ATM/ATR activation and is a key component of DNA damage response, but has also been implicated in several molecular processes involved in DNA structure modification and cell cycle progression^43^. We also observed increased clonogenic growth in HeLa cells with both DOX-induced expression of 4Ig-B7-H3-DD (confirming the above results), and a further increase from enhanced dimerization upon BB treatment, but importantly, only in the context of 4Ig-B7-H3-DD expression **(**Fig. 6f**)**. These data confirmed that tumorigenic signaling cascade activation and increased clonogenic growth occur upon 4Ig-B7-H3 homo-dimerization, and that this is not limited to a single biological system. Indeed, formation of 4Ig-B7-H3 dimers, rather than just increased expression or membrane localization alone, were responsible for the maximum observed tumorigenic functions. Thus, a model is proposed in which 4Ig-B7-H3 expression and dimerization drive the intracellular intrinsic (*cis*) activation of several signaling cascades that ultimately promote tumor cell growth and proliferation contributing to a pro-tumorigenic phenotype **(**Fig. 6g**)**.

## Discussion

B7-H3 is a known member of the B7-family of immune modulatory proteins that has gained considerable attention in recent years due to the high differential expression on many solid tumors compared to non-cancerous tissue or normal cells. Despite intensive research to understand its role as an immune checkpoint protein, aspects of B7-H3 functional biology and, in particular, how B7-H3 functions on the cell surface to elicit a tumorigenic response are still poorly understood. In the present work, we combined quantitative molecular imaging techniques in engineered biological systems to interrogate the nanoscale localization of 4Ig-B7-H3 isoforms and downstream effects of protein proximity (homo-dimerization) in fixed and live cells under physiological conditions, providing the first evidence of dimerization and functional consequences related to this PPI.

Previous data suggested a role for protein-dimerization of the B7-family of immune regulatory proteins to elicit an immune response. Herein, we sought to determine whether B7-H3 dimerized, and if dimerization structures related to tumor cell-autochthonous tumorigenic signaling and function *in cis*. The crystal structure of murine 2Ig-B7-H3 extracellular domain has been solved and provided the initial evidence of a potential dimerization interface^21^. While it was unclear if the dimerization interface observed in the crystal structure was due to a packing artifact that occurred during the crystallization process or functional, the structural components of the extracellular domain of B7-H3, namely the IgC-like domains, support the potential for dimerization or oligomerization. Members of the IgC family are components of immunoglobulin, cell surface and secretory glycoproteins, T-cell receptors, and MHC molecules, wherein the IgC domain is often involved in oligomerization and molecular interactions^44^. Molecular modeling of the human 4Ig-B7-H3 and 2Ig-B7-H3 extracellular domains using SWISS-PROT revealed two distinct patterns of potential dimerization, where the human 2Ig-B7-H3 isoform closely resembled the murine 2Ig-B7-H3 crystal structure; however, the human extracellular domain of 4Ig-B7-H3 modeled as a monomer with a distinct fold between the tandem repeats **(**Fig. 3a**)**, providing an alternative surface for a dimerization interface.

Our data indicated the presence of close proximity 4Ig-B7-H3 interactions (homodimers) in live cancer cells that conferred intrinsic tumorigenic signaling *in cis*, which contrasts from conventional views on B7-H3 functioning primarily as a monomer in immune-modulatory functions acting in trans. Using several inducible systems in which doxycycline readily titrated protein expression to levels near or below endogenous (physiological) expression levels, the results herein were consistent with 4Ig-B7-H3 proteins forming dimers and potentially higher order clusters on the cell membrane of cancer cells. While point mutations that disrupt dimerization may provide insight into the specific interacting domains, they can also be confounded by changes in expression, localization or processing of the native protein. Therefore, use of an chemically inducible dimerization system to interrogate the mechanism of increased tumorigenic signaling further allowed us to separate the functional roles of 4Ig-B7-H3 expression per se from dimerization in relation to tumorigenic signaling and tumor growth. BB-induced enhanced dimerization, even at very low exogenous expression levels of 4Ig-B7-H3, resulted in activation of AKT signaling, the Jak/STAT pathway and modulators of the HIF1α and NF-κβ pathways. Note that the chemically-enhanced dimerization system was linked *in cis* within the cytosolic domain of the 4Ig-B7-H3 fusion construct, and thus, *cis* acting cascades would be the most direct signaling pathway. Although induced expression of 4Ig-B7-H3 alone in this system increased tumorigenic signaling across several key pathways over the null baseline, it was further boosted significantly and consistently upon enhanced dimerization, indicative of an enhanced tumorigenic role of 4Ig-B7-H3 dimers compared to that of 4Ig-B7-H3 monomers at equilibrium. The most notable increase was phosphorylation of HSP27 at S78/S82, with known anti-apoptotic functions. Further investigations are required to address in detail whether dimer formation is necessary for 4Ig-B7-H3-mediated basal tumorigenic signaling and to determine the functional consequences of altering 4Ig-B7-H3 dimerization on cancer cell intrinsic and extrinsic signaling pathways, particularly in the context of immune modulation. Given the role of ligand-receptor interactions between tumor cells and immune cells, it is perhaps not difficult to imagine a role for *cis*-dimerization as a method to regulate receptor binding or activation in trans. More broadly, future studies should address the potential immunomodulatory mechanisms afforded by the discovery of B7-H3 dimerization states.

The data presented herein further suggested that physiologic dimerization was, at least in part, mediated by the extracellular domain of 4Ig-B7-H3 because we observed dimerization by size exclusion chromatography using purified active recombinant protein that was truncated proximal to the transmembrane and intracellular C-terminus domains. In addition, by using functionally active protein as determined by T-cell proliferation assays developed and performed as part of the protein quality control (performed by R&D Systems), our data further supported the significance of 4Ig-B7-H3 dimerization to functionally elicit a tumorigenic phenotype. Future studies to determine the mechanisms of dimerization and the key dimerization domains through mutational analysis will need to be addressed, but the role of scaffolding proteins, or additional dimerization interfaces should not be ruled out based on existing data. Mutationally defining the dimerization interface(s) could provide targeted regions for therapeutic development to interfere with 4Ig-B7-H3 dimerization. In addition, further biochemical, microscopy and structural studies are warranted to interrogate the role of isoform-specific or non-specific dimerization. Given the conflicting functional data obtained for B7-H3 between murine and human species in which the dominant isoform differs between 2Ig and 4Ig, respectively, additional studies across species will be required to decipher the role of dimerization on isoform-specific functional consequences on both the tumor intrinsic functions (*cis*) as well as roles to modulate tumor extrinsic function, including the immune system. Without a known receptor(s) identified on the immune cell, the impact of B7-H3 dimerization *in cis* or in trans (which remains an open possibility) to influence cell extrinsic function remains an unexplored area of impact.

Compared to baseline, we have demonstrated inducible proximity of 4Ig-B7-H3 pairs, most consistent with homo-dimerization, as a potential novel mechanism for regulating the biological activities of B7-H3. Our findings have noteworthy implications for both the basic understanding of B7-H3 biology and pharmaceutical targeting of B7-H3. Although the correlation of poor prognosis with expression has largely implicated a pro-tumor role of B7-H3 expression across many tumor types, there is emerging evidence that B7-H3 expression is also linked to therapeutic resistance to chemotherapy, and increased metastasis^6,45^. While ongoing studies are leveraging tumor cell-expression of B7-H3 for therapeutic intervention (CAR-T therapy, anti-B7-H3-ADCs, anti-B7-H3 radiotheranostics)^46–50^, we propose an additional approach focused on disrupting 4Ig-B7-H3 dimerization to inhibit tumor growth and new therapeutic agents that accomplish this might prove effective in the control of B7-H3-driven tumor progression and resistance. Although inhibitory anti-B7-H3 antibodies have made significant translational progress, their ability to impact 4Ig-B7-H3 homodimerization remains unknown. These findings further support B7-H3 as a bona fide therapeutic target for cancer, and studies to identify modulators of B7-H3 dimerization as an alternative interventional strategy are underway capitalizing on the high-throughput potential of using the 4Ig-B7-H3 split luciferase complementation assay.

## Materials and Methods

### Antibodies and reagents

Anti-β-actin (4970 L), anti-HA-tag (3724 S), anti-GFP-tag (2956 S) antibodies were purchased from Cell Signaling Technology. Anti-GAPDH (G9545) was purchased from Sigma-Aldrich. Anti-B7-H3 antibody was produced in house (MIL33B) or purchased from R&D Systems (AF1397). Secondary antibodies were purchased from BioRad Laboratories: goat anti-Mouse (L005680), goat anti-Rabbit (10000045946), rabbit anti-Goat (L006330A). The U2OS-4Ig-B7-H3 cell line was generated using the ReBiL 2.0 plasmid system and cloned using Gibson assembly to express 4Ig-B7-H3 fused with a C-terminal HA-tag linker to N-Luciferase or C-Luciferase. Vector-mEGFP and CD276-mEGFP plasmids were generated by Genecopoeia (Rockville, MD) using CD276 ORF expression clone (NM_00102736.1) or empty control vector for pReceiver-M98 (EX-Neg-M98). Tet-On inducible-homo-dimerization system was purchased from Clontech Laboratories Inc. (Mountain View, California; Catalog: 635068) and 4Ig-B7-H3 was cloned into the construct using PCR-based amplification and In-Fusion assembly according to the manufacturers protocol. CRISPR/Cas9-directed *CD276* knockout HeLa and SKOv3-ip-FLuc cell lines were engineered using a dual plasmid gRNA and Cas9 plasmid system (Santa Cruz Biotechnology) and clonally expanded from single cells. Gene knockout was confirmed by qRT-PCR and Western blot analysis.

**Table:** 

Antibody Target	Source	Catalog #	Dilution	Application
B7-H3 (AF1397)	R&D Systems	AF1397	1:2000	WB
B7-H3 (MIL33B)	In house	N/A	1:1000-5000	WB
GAPDH (G9545)	Sigma Aldrich	G9545-100UL	1:10,000	WB
B-Actin (13E5)	Cell Signaling Technology	4970	1:10,000	WB
HA-tag (C29F4)	Cell Signaling Technology	3724	1:1000	WB
GFP-tag (D5.1)	Cell Signaling Technology	2956	1:1000	WB, Flow
Goat-anti-mouse	BioRad	L005680	1:10,000	WB
Goat anti-Rabbit	BioRad	10000045946	1:10,000	WB
Rabbit anti-Goat	BioRad	L006330A	1:10,000	WB
B7-H3 (AF1397)	R&D Systems	AF1397	1:400	IF, IHC
B7-H3 (MIL33B)	In house	N/A	1:200-500	IF, IHC
B7-H3 (D9M2L)	Cell Signaling Technology	14058	1:100	IHC

### Cell culture and transfection conditions

Cells were maintained using standard aseptic techniques in a humidified atmosphere of 5% CO_2_. Human cell lines were routinely checked for mycoplasma contamination and routine short tandem repeat DNA fingerprinting was performed to ensure proper cell line identification. All cell lines were maintained at less than 15 passage levels, where the majority of experiments were performed between passage numbers 5-10 and when the cells were in a linear growth phase. U2OS ReBiL cell lines were routinely grown in DMEM supplemented with 2mM L-glutamine, nonessential amino acids, and 10% tetracycline-free fetal bovine serum. Gene expression was induced by adding 0.1 µg/mL doxycycline (Dox) as indicated. HeLa cell lines and derivatives were grown in DMEM high glucose media supplemented with 10% FBS and 1% L-Glutamine. SKOv3-ip-FLuc cell lines and derivatives were grown in McCoy’s 5 A media supplemented with 10% FBS and 1% L-Glutamine. Transient transfection was performed using MegaTran 1.0 (Origene, #TT200005) according to the manufacturer’s protocol. For transfection of a 6-well plate, 3 μg of DNA was combined with 9 μL of transfection reagent in 250 μL of OPTIMEM and allowed to incubate at room temperature for 10 min before being added to the cultured cells. When co-transfection was performed, DNA concentrations were reduced to 1.5 μg of each plasmid. Lentiviral particles were purchased from GeneCopoeia (CD276 LLP-Z3060-LV105-100) or Cellomics Technology (Firefly luciferase-CMV) and transduction was performed when cell densities reached ~70% confluence. Lentiviral infection was performed with a target MOI = 1.5-3. CD276 re-expression cell lines were stably expanded without further selection maintaining a heterogenous population as shown in Supplemental Fig. 1. FLuc lines were subjected to neomycin selection and reporter gene expression was assessed using bioluminescence imaging on stable clones.

### Cell proliferation assay

Changes in cancer cell growth with or without 4Ig-B7-H3 expression were determined using clonogenic and sulforhodamine B (SRB) assays^51^. Clonogenic assays were performed using U2OS-4Ig-B7-H3, HeLa (WT, KO, and rescue lines) and SKOv3-ip-FLuc (WT, KO), where 2000-5000 cells/well were cultured with or without Doxycycline (if applicable) for 72 h. Cells were then allowed to grow under normal tissue culture conditions in a clonogenic fashion for two weeks, and stained with Coomassie blue, before the colonies were quantified. SRB short term cell viability assays were performed using HeLa and SKOv3-ip-FLuc WT and KO cells (5 × 10^3^) and U2OS-4Ig-B7-H3 cells cultured with or without doxycycline for 72 h. Cells were washed, and fixed with 30% TCA at 4°C, and incubated for 30 min at room temperature with 0.4% SRB in 1% acetic acid. SRB dye was solubilized with Tris base for 10 min with gentle agitation. Plates were read with a microplate-reader (Biotek synergy 2, Tecan) at 510 nm.

### Western blotting

Cell lysates were prepared as indicated following incubation in lysis buffer (50 mM Hepes, pH 7.0, 150 mM NaCl, 1.5 mM MgCl_2_, 1 mM EGTA, 10 mM NaF, 10 mM sodium pyrophosphate, 10% glycerol, 1% Triton X-100) plus protease and phosphatase inhibitors (1 mM PMSF, 10 µg/mL leupeptin, 10 µg/mL aprotinin and 1 mM Na_3_VO_4_). Cells were lysed for 30 min on ice, and then centrifuged at 17,000 x g for 30 min at 4°C. Protein concentration was assessed using a bicinchoninic acid (BCA) protein assay (ThermoScientific, #23225). Equal amounts of protein were separated by 8-16% SDS-PAGE, transferred to PVDF membranes and subjected to Western blotting using an ECL chemiluminescence reagent (BioRad, #NEL105001).

### Immunofluorescent staining

Tumor cells (3 × 10^4^) were seeded in chamber slides and treated as specified. Cells were fixed with 4% paraformaldehyde and permeabilized with 0.5% Triton X-100. Cells were washed with PBS, blocked with 5% BSA/PBS, followed by incubation with the primary antibody (overnight at 4°C or rt for 2 hr). After washing, cells were incubated with secondary antibodies (rt for 1 hr) conjugated with Alexa Fluor 488 or 594 (Molecular Probes, #A11017, #A11020, #A11070, #A11072), mounted and examined using a fluorescence microscope (Nikon TiE microscope with an XM10 camera).

### Size exclusion chromatography

Using a Superdex 200 10/300 column with PBS + 0.1 M NaCl + 0.05% NaN3 as eluent (flow 0.5 ml/min), a standard curve was created using proteins of known molecular mass at 0.5 mg/ml concentration and recording their elution times (Gel Filtration Markers Kit for Protein Molecular Weights 12-200 kDa, Millipore Sigma (MWGF200)). Experimental molecular weights derived by plotting the logarithm of the molecular weight (LogMW) against the partition coefficients (K_av_), which were defined by V_e_-V_0_/V_t_-V_0_, where V_e_ represented elution volume, V_0_ represented void volume, and V_t_ represented total volume.

### Detection of dimerization by fluorescence lifetime imaging analysis

Fluorescence lifetime imaging was performed at the Center for Advanced Microscopy, a Nikon Center of Excellence, Department of Integrative Biology & Pharmacology at McGovern Medical School, UTHealth Houston. Forster resonance energy transfer-fluorescence lifetime imaging microscopy (FRET-FLIM) experiments were performed using the Nikon A1 basic confocal system installed with a PicoQuant FLIM LSM upgrade kit. Data acquisition and analysis were performed using the PicoQuantSymPho Time 32 software. Transiently transfected cells were fixed using 4%PFA at room temperature for 10 min and washed briefly with PBS (3X) for 5 min and kept shielded from light until analyzed at the Center for Advanced Microscopy. For fluorophore excitation, a 483 nm pulsed laser with an excitation frequency of 40 MHz was applied. This was followed by FLIM measurement of the average amount of time for the fluorophore to remain in the excited state using the PicoQuant SPAD detection unit and FF01 520/35-25 filter. The instrument response function (IRF) was determined at the beginning and end of each imaging session using a Convallaria sample according to the manufacturers protocol. Photon count rate was kept under 10% of the pulse rate, and enough frames were acquired to obtain the cumulative signal intensity of at least 10^4^ photons. Fluorescence decay curves were fitted to a bi-exponential reconvolution function adjusted to the IRF and the average lifetime was calculated and represented in the FLIM images as τ. The lifetime of 4Ig-B7-H3-mEGFP was measured and compared to Vector-mEGFP, following adjustment of transfection conditions such that each protein was expressed at similar levels as determined by both Western blot analysis and surveying the imaging field with epifluorescence microscopy. All measurements were taken from whole-field images of cells, and at least 10 measurements were taken for each analysis over 3 biological replicates.

### ReBiL assay of B7-H3 multimerization

Cells were seeded in 96-well or 384-well black wall, clear bottom plates and treated with doxycycline (100 ng/mL) to induce gene expression. 24-48 h following gene expression, cells were washed briefly with 1X PBS and D-luciferin (200 µM) was added in phenol red-free DMEM/F12 medium supplemented with MEBSS. Following incubation at 37 °C, bioluminescence was initiated by addition of 100 μL substrate solution containing D-luciferin dissolved in PBS (final concentration = 300 µM). Bioluminescence imaging was performed using an IVIS100 or IVIS Spectrum instrument (FOV C, 1-5 sec exposure, open filter, medium binning). Bioluminescence microscopy of U2OS-4Ig-B7-H3 live cells was performed on a Nikon TiE inverted microscope (Nikon Instruments, Melville, NY, USA) equipped with a back-illuminated 1024 × 1024 pixel CCD with a 13 µm pixel pitch (iKon-M 934; DU934P-BEX2-DD, Andor Inc/Oxford Instruments (Belfast, Northern Ireland), which was air cooled to −85 °C during normal operations with deep depletion fringe suppression and anti-reflective coating^52^. When acquiring bioluminescence images, the camera was read out at 50 kHz, 4X gain, and binning 2 to minimize read noise and maximize sensitivity. Cells were maintained at 37 °C in a humid environment for the duration of imaging (10X objective, 20 min acquisition, open filter).

### Reverse phase protein array

RPPA as performed in the MD Anderson CORE laboratory has been described previously^53,54^ and was used to quantify endogenous and phosphorylated protein content for nearly 499 protein targets. Briefly, cells were lysed and concentrations normalized to 1 μg/μL concentration using bicinchoninic acid assay and boiled with 1% SDS. Protein extracts were diluted 2-fold in five serial dilutions (undiluted to 1:16) and spotted onto nitrocellulose-coated slides. These extracts are arranged in a 4 × 12-pin-11 × 11 subarray that accommodate 1056 serially diluted samples plus replicate vertical controls on each slide. The protein of interest was detected by probing with a specific antibody, amplifying the signal via a tyramide amplification system, and visualized via DAB colorimetric reaction. Each slide was probed with one antibody. The detection system used was a GenPoint-based staining kit from Agilent. Digital images of slides were obtained by scanning on a Huron TissueScope scanner producing 16-bit TIFF files. Spot intensities from the TIFF files were determined via Array-Pro Analyzer software. Mean net intensities of spots from all 1056 samples were used for curve-fitting for each slide (RPPA Super Position and Concentration Evaluation, aka SPACE). Relative protein level for each sample was determined by converging its sample dilution series to one point (EC_50_) and interpolating in RPPASPACE. RPPASPACE was developed by the UT MD Anderson Cancer Center Department of Bioinformatics and Computational Biology. Cell lysates were analyzed in the same batch, but samples were acquired from two independent biological replicate experiments. Relative protein levels for each sample were determined by interpolation of dilution curves from the “standard curve” (supercurve) of the slide for each particular antibody. All data were presented as fold-change 4Ig-B7-H3 KO compared with baseline (wildtype HeLa or SKOv3-ip cells). Positive fold-change was calculated by dividing each linear value (>1.0) with average control linear values for each antibody tested, while negative fold-change (for linear values < 1.0) was calculated using the following formula: ([−1/linear fold-change]/2) as a log 2.0 value.

### Phospho-kinase signaling array

3 Χ 10^6^ cells/well were seeded into a 6-well dish and allowed to adhere overnight. Doxycycline was added to one well for 48 h to induce 4Ig-B7-H3 expression and dimerization. Cell lysates were obtained following the manufacturers protocol for the Human Phospho-kinase antibody array (R&D Systems; Catalog: ARY003B). Site-specific phosphorylation of 43 kinases and 2 related total proteins were performed and analyzed. Relative changes in protein phosphorylation and expression levels were compared between DOX- and DOX+ lysates, and the assay was performed in biological duplicate, with the average of both experiments being presented.

### Animal models and in vivo studies

All animal procedures were approved by The University of Texas M.D. Anderson Cancer Center Animal Study Committee (IACUC). We have complied with all relevant ethical regulations for animal use. Six-week-old athymic nude mice (strain *nu*/*nu*, Charles River) were anesthetized (1.5% isoflurane inhalation) during tumor cell implantation. 5 × 10^6^ HeLa cervical cancer cells were injected subcutaneously in the rear flank in 100 μL serum-free media. Tumors were measured (L x W) twice weekly using a caliper and mouse weight was monitored throughout the study. Tumor volume was calculated by the modified ellipsoidal formula: V = ½ (Length x Width^2^). Six week-old athymic nude mice (strain *nu*/*nu*, Charles River) were injected intra-peritoneally with 1.0 × 10^6^ SKOv3-ip-FLuc cells/mouse in an orthotopic fashion. SKOv3-ip-FLuc cells stably express firefly luciferase producing constitutive bioluminescence signals in the presence of substrate. I.P. injection of D-luciferin (prepared at 30 mg/mL in PBS) was used to noninvasively monitor tumor burden. Mice were imaged 10 min post-D-Luciferin injection using the PerkinElmer IVIS Spectrum Imaging System weekly beginning at baseline and following I.P. orthotopic injection of ovarian cancer cells.

### Statistics and Reproducibility

Data were expressed as mean ± standard deviation, unless otherwise specified. Statistical significance was analyzed using a two-tailed students *t* test, Wilcoxon-rank log test, or One-way and two-way ANOVA for multiple group comparisons. *P* values < 0.05 were considered statistically significant and denoted by a single asterisk. *P* values < 0.01 were denoted by a double asterisk. Experiments were performed in biological triplicate (three independent experiments conducted over independent timeframes) unless otherwise specified and often included multiple technical replicates in each experiment (same conditions within a given experiment). Specific experimental details are included in the figure legends.

### Reporting summary

Further information on research design is available in the Nature Portfolio Reporting Summary linked to this article.

### Supplementary information

Supplemental information

Description of Additional Supplementary Files

Supplemental Data 1

Supplemental Data 2

Supplemental Data 3

Reporting Summary

## Data Availability

All source data is contained within the manuscript and supplemental information and source data is contained within Supplemental Data 3. Uncropped blots are provided in Supplemental Fig. 7.
