# Supplementary material for: Dimerization of the 4Ig isoform of B7-H3 in tumor cells mediates enhanced proliferation and tumorigenic signaling
Source: Commun Biol. 2024 Jan 5;7:21. doi: 10.1038/s42003-023-05736-8 (PMC10770396; doi:10.1038/s42003-023-05736-8)
Supplement: Supplementary file 1 — Supplemental information [file 42003_2023_5736_MOESM1_ESM.pdf]

Supplemental Information

Dimerization of the 4Ig Isoform of B7-H3 in Tumor Cells Mediates Enhanced Proliferation and Tumorigenic Signaling

Margie N. Sutton[1], Sarah E. Glazer[1], Riccardo Muzzioli[1], Ping Yang[1], Seth T. Gammon[1], David Piwnica-Worms[1]*

[1]Department of Cancer Systems Imaging, The University of Texas M. D. Anderson Cancer Center, Houston, Texas 77030, USA

Email: dpiwnica-worms@mdanderson.org

Author Contributions: M.N.S. and D.P.W. designed the study. M.N.S., S.T.G., S.E.G., and R.M. carried out experiments. D.P.W. provided resources and reagents. M.N.S., S.T.G., S.E.G., R.M., and D.P.W. analyzed data, and M.N.S. and D.P.W. wrote the manuscript. All authors edited and approved the manuscript.

Competing Interest Statement: The authors declare no competing interests.

Keywords: B7-H3, CD276, protein dimerization, split-luciferase complementation, bioluminescence, live cell fluorescence microscopy, FLIM

*Corresponding author:
David Piwnica-Worms, M.D., Ph.D.
1881 East Road, Unit 1907
Houston, TX 77054
Email address:
dpiwnica-worms@mdanderson.org

Supplemental Figure 1

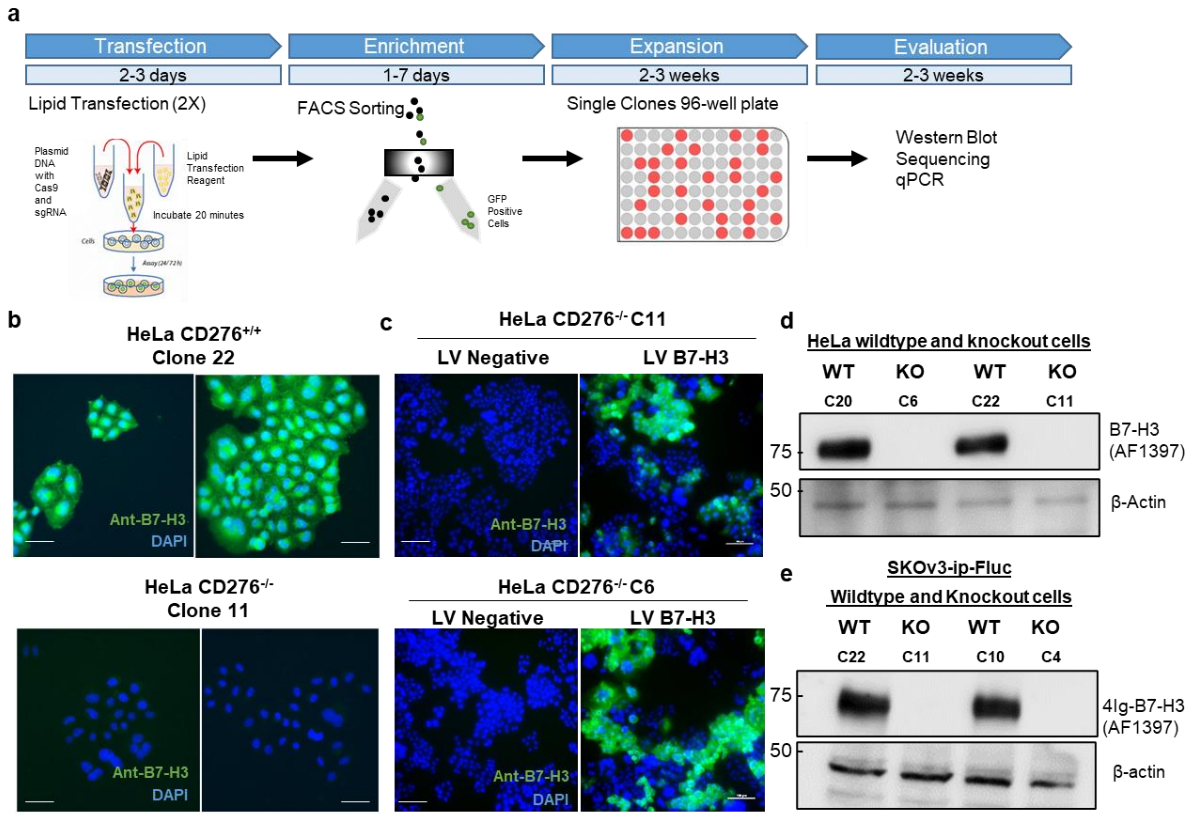

Supplemental Figure 1. Generation of B7-H3 knockout and rescue HeLa cell lines. a. Scheme depicting the protocol and workflow to generate CRISPR/Cas9-based *CD276* knockout single clones. **b.** Immunofluorescence staining of endogenous B7-H3 using anti-B7-H3 antibody (green) and DAPI (blue) in WT (clone 22) and B7-H3 KO (clone 11) cells. **c.** Immuno-fluorescence staining of 4Ig-B7-H3 rescue expression (green; DAPI in blue) in pooled clones generated following lentiviral transfection of 4Ig-B7-H3. HeLa *CD276*$^{-/-}$ knockout cells (clones 11 and 6) were transfected with either lentiviral negative control vectors or 4Ig-B7-H3. **d-e.** Western blot analysis of 4Ig-B7-H3 expression and β-actin of several single clone cancer cell lines; HeLa **(d)** and SKOv3-ip-FLuc **(e)**.

Supplemental Figure 2

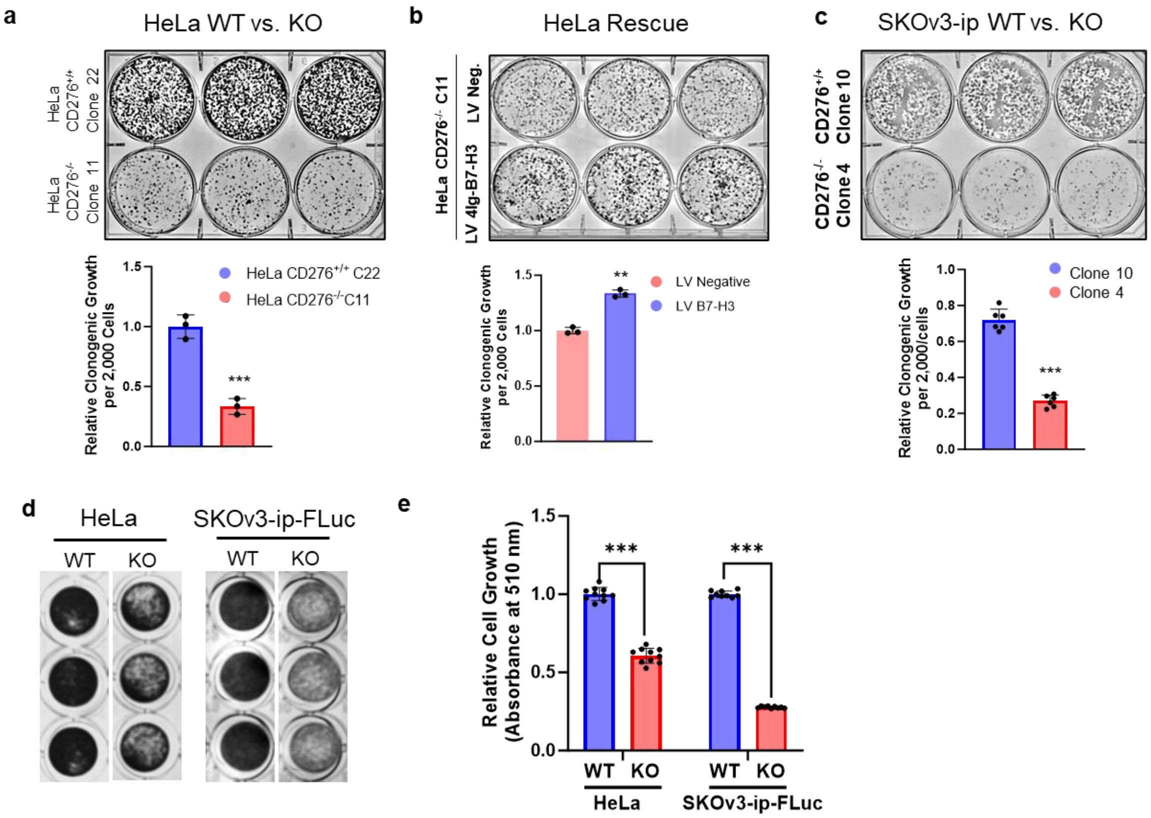

Supplemental Figure 2. Knockout of B7-H3 reduces clonogenic growth and tumorigenic signaling in a second set of gynecological cancer cells. A second set of HeLa wildtype and knockout clones with matched rescue cell lines, as well as SKOv3-ip-FLuc paired WT and KO clones were used to assess B7-H3-dependent growth as described in Figure 1. **a.** 2,000 HeLa wildtype (Clone 22) or *CD276* knockout cells (Clone 11) were seeded and allowed to grow for 10 days, changing the media every 72 hours. **b.** HeLa $CD276^{-/-}$ cells engineered to re-express 4Ig-B7-H3 (LV 4Ig-B7-H3) or a negative vector control (LV Neg) were used to perform a clonogenic assay as described above. 2,000 cells were seeded and allowed to grow for 10 days, changing the media every 72 hours. **c.** 2,000 SKOv3-ip-FLuc wildtype (Clone 10) or *CD276* knockout cells (Clone 4) were seeded and allowed to grow for 10 days, changing the media every 72 hours. **a-c.** Clonogenic growth was quantified using ImageJ and represented in the bar graph (mean ± SD). The experiment was performed in technical triplicate with three biological replicates, ***$p<0.001$. **d.** Short-term growth assay. 5,000 HeLa or SKOv3-ip-FLuc (WT or KO) cells were seeded in a 96-well plate and allowed to grow for 72 hours prior to fixation and staining with sulforhodamine B (SRB). Plate images were captured under visible light at 1 second exposure. **e.** SRB dye was released using Tris base, and absorbance at 510 nm was measured. Relative growth for each cell line was determined and normalized to WT growth (mean ± SD). The experiment was performed in technical triplicate with three biological replicates, ***$p<0.001$.

Supplemental Figure 3

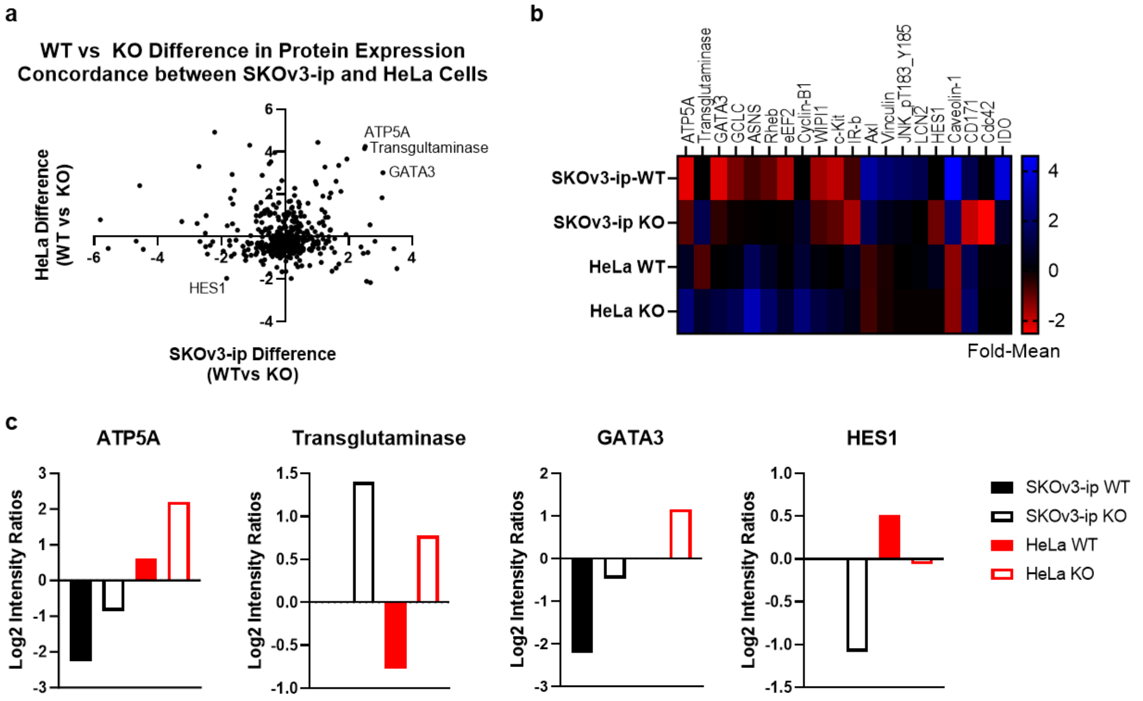

Supplemental Figure 3. Concordant changes in RPPA protein expression between WT and KO genotype for both SKOv3-ip and HeLa cells. a. SKOv3-ip cell difference between CD276-/- protein expression and WT protein expression (x-axis) for 499 proteins probed by RPPA analysis was compared to the difference between expression of HeLa KO and WT proteins for the same protein (y-axis). Concordant changes that resulted in increased protein expression upon knockout are found in the upper right quadrant while significant decreases upon loss of B7-H3 are found in the lower left quadrant. **b.** Relative expression levels (median centered) for the top and bottom 10 most significantly changed proteins when the mean difference in expression was calculated for each cell line. **c.** Log2 Intensity ratios for each concordant protein as measured in SKOv3-ip WT (black solid), SKOv3-ip KO (black open), HeLa WT (red solid) and HeLa KO cells (red open).

Supplemental Figure 4

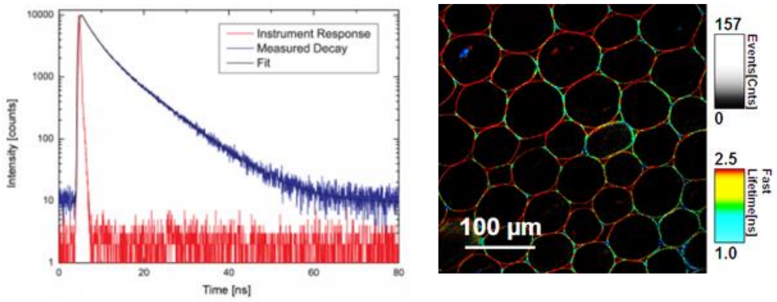

Supplemental Figure 4. Instrument response function (IRF) determined using lifetime fluorescence imaging of Convalaria. Measured decay curves (left) and pseudocolored lifetime imaging of the specimen (right) were used to set the IRF at the beginning and end of each imaging session.

Supplemental Figure 5

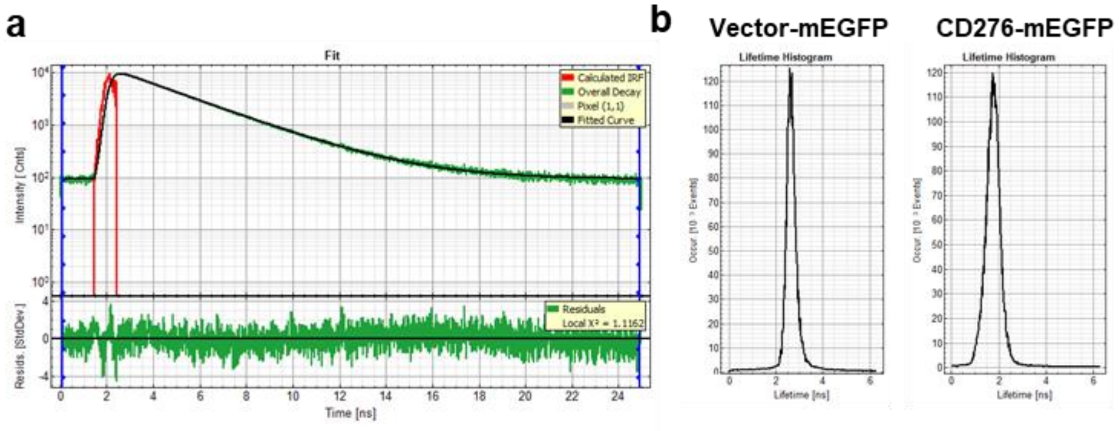

Supplemental Figure 5. Exponential Decay fitting of mEGFP lifetime and histogram distribution of lifetimes captured for homo-FRET-FLIM analysis of B7-H3 dimerization. **a.** Exponential decay curve fitting obtained using SymphoTimes software. **b.** Histogram analysis of mEGFP lifetimes representative of samples transfected with Vector-mEGFP or CD276-mEGFP.

Supplemental Figure 6

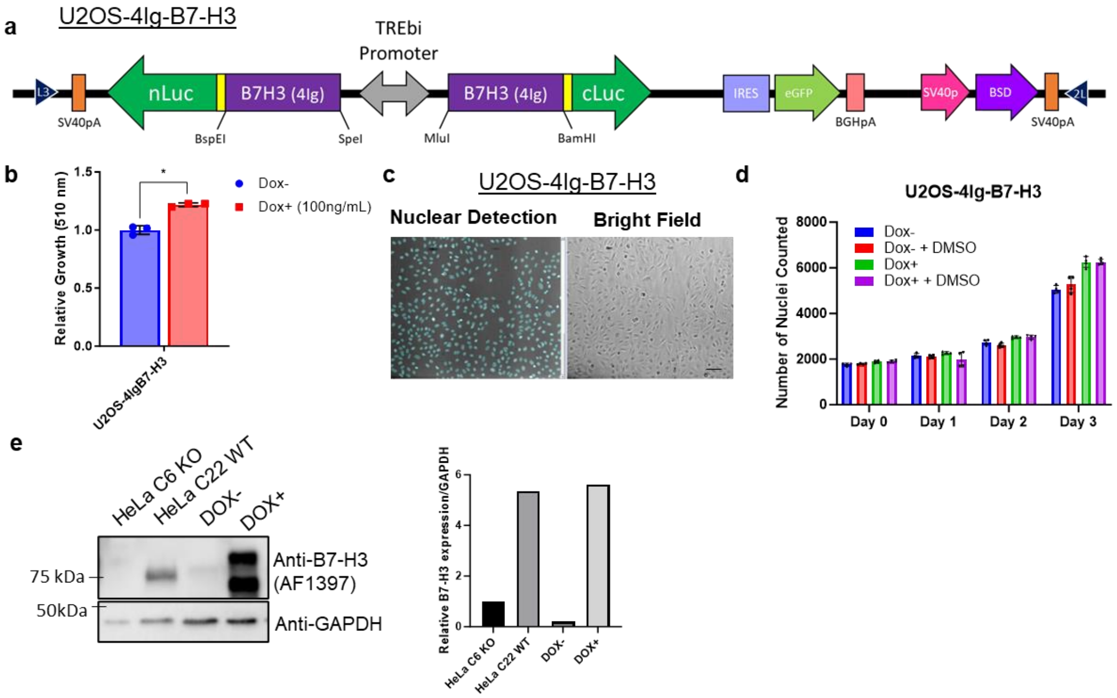

Supplemental Figure 6. Generation of U2OS-4Ig-B7-H3 ReBiL inducible cell line. **a.** Model depiction of the plasmid construct containing a TRE bidirectional doxycycline inducible-promoter. **b.** SRB assay of short-term proliferation and viability of U2OS-4Ig-B7-H3 ReBiL cells with and without doxycycline for 72 hours. **c.** Nuclear detection and automated counting of nuclei, quantified in **(d),** for U2OS-4Ig-B7-H3 ReBiL cells treated with or without doxycycline or DMSO. **e.** Western blot expression of endogenous 4Ig-B7-H3 in HeLa WT or KO cells, compared to expression in U2OS-4Ig-B7-H3 ReBiL cells treated with or without doxycycline for 72 hours (left). PDVF membrane was probed using anti-B7-H3 antibody (AF1397) and anti-GAPDH and the relative intensities for 4Ig-B7-H3 (endogenous vs. 4Ig-B7-H3-nLuc/cLuc) were quantified via ImageJ (right).

Supplemental Figure 7. Full gels for all Western blot experiments presented within the manuscript and supplemental figures.

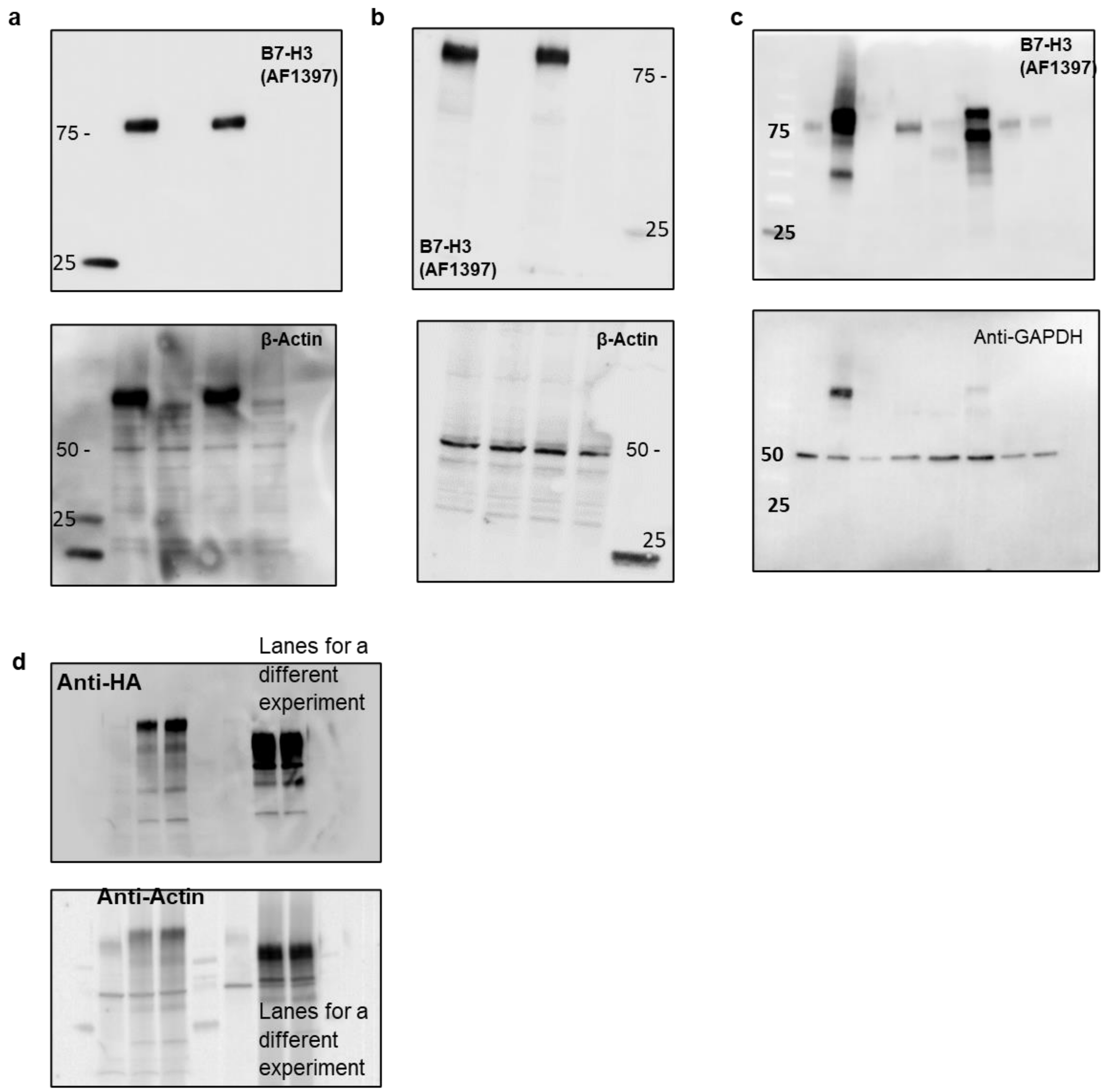

Supplemental Figure 7. Full gels for all Western blot experiments presented within the manuscript and supplemental figures. Full blots from Supplemental Figure 1d **(a)** and 1e **(b).** Full blots from Supplemental Figure 6 **(c)**. Full blots from Figure 5 **(d)**.

Supplemental Table 1. SEC predicted molecular weights for human 4Ig-B7-H3 and murine 2Ig-B7-H3. Table defining the predicted molecular weight and characteristics of murine and human B7-H3 used in subsequent size exclusion chromatography (SEC) analysis.

Human 4Ig-B7-H3 Recombinant Protein

Gly 27- Thr 461 with C-term 10-his tag

Predicted MW: 48 kDa

SDS Page MW: 78-90 kDa (reducing conditions)

	Kav	Log MW	MW (kDa)
hB7-H3	0.89	5.45	279.3
hB7-H3	1.11	5.23	170.2

Murine 2Ig-B7-H3 Recombinant Protein

Val 29- Phe 244 with C-term 10-his tag

Predicted MW: 24.8 kDa

SDS Page MW: 38-45 kDa (reducing conditions)

	Kav	Log MW	MW (kDa)
mB7-H3	1.25	5.10	126.4
mB7-H3	1.51	4.85	71.5

Supplemental Table 2. Log2 transformed raw data from kinase array presented in the heat map in Figure 6d.

	DOX-		DOX+		DOX- BB		DOX+ BB	
CREB S133	-5.45236	-5.7409	-7.36361		-5.51056	-5.57677	-4.12424	-4.45777
Fgr Y412	-8.21004	-8.23286	-7.25253	-7.96759	-7.16463	-6.35982	-4.51167	-4.90198
JNK 1/2/3 T183/Y185, T221/Y223	-5.54133	-5.38467	-6.17407	-6.16939	-5.71535	-5.47227	-4.65494	-5.26645
p38a T180/Y182	-2.47521	-2.5624	-2.9809	-2.87917	-2.79521	-2.84027	-2.96754	-3.22127
Stat2 Y689	-5.45558	-4.96998	-5.49174	-6.23037	-6.73989	-5.92244	-4.17679	-4.24283
B-Catenin	-6.30278	-6.05995	-5.14244	-6.41575	-6.24879	-7.31181	-4.96953	-5.59904
EGFR Y1086	-9.88717			-10.1355	-10.2609	-6.57251	-6.61359	-5.92165
GSK-3a/b S21/S9	-6.64805	-6.87612	-9.05582	-7.2466	-6.08912	-5.5723	-6.72272	-6.05218
PDGF Rb Y751	-5.04745	-5.39289	-7.07787	-6.67109	-5.53831	-5.29891	-5.36843	-5.12603
Stat5a/b Y694/Y699	-2.50108	-2.6235	-2.89384	-2.86414	-2.75489	-2.68042	-3.59116	-3.30277
eNOS S1177	-6.85075	-6.66625	-6.81799	-6.98503	-6.12512	-6.08177	-5.06593	-4.95246
GSK-3B S9	-2.45627	-2.55452	-2.88904	-2.97818	-2.03927	-2.10599	-2.91522	-2.85352
Lyn Y397	-6.85826	-6.73435	-5.65225	-5.84411	-5.60191	-5.85701	-5.51167	-5.21845
PLC-g1 Y783	-5.56204	-5.69451	-6.05333	-5.92472	-5.03174	-5.12835	-4.49877	-4.45672
WNK1 T60	-3.9976	-4.17264	-4.51296	-4.53266	-3.72845	-3.7464	-3.11619	-3.1941
ERK1/2 T202/Y204, T185/Y187	-7.39193	-7.58173	-8.77612	-9.38854	-6.70953	-6.95036	-5.01546	-5.13355
HSP27 S78/S82	-7.92911	-7.75321	-10.8126		-6.46536	-6.68216	-4.69656	-4.82198
MSK1/2 S376/S360	-5.92594	-6.05294	-6.54517	-6.53885	-5.58104	-5.72532	-3.63333	-3.52299
Src Y419	-5.09271	-5.1402	-5.23699	-5.05582	-4.80866	-4.90211	-4.75364	-4.56487
Yes Y426	-5.58008	-5.54976	-5.02079	-5.05841	-5.06067	-5.18973	-4.38672	-4.2268
AKT 1/2/3 T308	-5.65654	-5.78317	-5.86103	-5.78524	-6.64674	-6.59088	-4.60795	-4.7939
Chk-2 T68	-5.15998	-5.20833	-4.72692	-4.75941	-5.49193	-5.45349	-3.87096	-3.94311
p53 S15	-6.42749	-6.35662	-6.13868	-6.14785	-7.88923	-7.32864	-7.78595	-8.11819
p70S6kinase T389	-5.3221	-5.52809	-5.42056	-5.50066	-6.09433	-5.99751	-4.73113	-4.80551
PYK2 Y402	-5.39672	-5.48597	-5.93298	-6.07077	-6.48319	-6.0413	-6.13057	-6.03032
Stat1 Y701	-5.77052	-5.70162	-6.28535	-6.2679	-6.67212	-6.33935	-6.3673	-6.31697
Stat6 Y641	-5.85425	-5.71202	-5.27693	-5.3476	-5.96254	-5.84204	-4.42365	-4.52966
AKT1/2/3 S473	-6.50675	-6.52045	-6.12049	-6.16971	-7.03846	-7.05909	-7.08216	-7.09217
c-Jun S63	-6.164	-6.10731	-5.67565	-5.91982	-6.55436	-6.62209	-5.5745	-5.67794
p53 S46	-5.32702	-5.53624	-4.84162	-5.0385	-5.78317	-5.64516	-5.14566	-5.17282
p70S6kinase T421/S424	-6.32343	-6.48875	-6.52776	-6.86598	-6.91755	-6.88342	-5.25374	-5.33189
RSK1/2 S221/S227	-5.14224	-5.28242	-5.25898	-5.37626	-5.63358	-5.80632	-4.8427	-4.7858
Stat3 Y705	-5.93677	-6.01019	-6.17116	-6.34567	-6.4321	-6.86145	-4.86128	-4.9897
HSP60	-6.32331	-6.5568	-5.33102	-5.48332	-6.11169	-6.07427	-6.87307	-6.83091
p53 S392	-5.43023	-5.4077	-6.17074	-6.08745	-5.9015	-5.71892	-5.11669	-4.92034
PRAS40 T246	-4.11639	-4.0852	-4.90777	-4.69555	-4.36727	-4.15313	-3.9366	-3.85797
RSK1/2/3 S380/S386/S377	-4.37674	-4.28997	-5.44354	-5.32771	-4.60345	-4.52374	-4.03136	-3.92369
Stat3 S727	-3.54997	-3.46734	-3.94064	-3.87449	-4.09027	-4.03751	-4.09318	-3.99465
