# Supplementary material for: Dimerization of the 4Ig isoform of B7-H3 in tumor cells mediates enhanced proliferation and tumorigenic signaling
Source: Commun Biol. 2024 Jan 5;7:21. doi: 10.1038/s42003-023-05736-8 (PMC10770396; doi:10.1038/s42003-023-05736-8)
Supplement: Supplementary file 2 — Description of Additional Supplementary Files [file 42003_2023_5736_MOESM2_ESM.pdf]

Description of Additional Supplementary Files

File name: Supplemental Data 1
Description: Source Data for all graphs contained in the manuscript.

File name: Supplemental Data 2
Description: Full Blot images contained in the manuscript.

File name: Supplemental Data 3
Description: All source data behind the graphs in the figures.
