# Supplementary material for: Dimerization of the 4Ig isoform of B7-H3 in tumor cells mediates enhanced proliferation and tumorigenic signaling
Source: Commun Biol. 2024 Jan 5;7:21. doi: 10.1038/s42003-023-05736-8 (PMC10770396; doi:10.1038/s42003-023-05736-8)
Supplement: Supplementary file 6 — Reporting Summary [file 42003_2023_5736_MOESM6_ESM.pdf]

nature portfolio

Corresponding author(s): David Piwnica-Worms

Last updated by author(s): 12/14/2023

Reporting Summary

Nature Portfolio wishes to improve the reproducibility of the work that we publish. This form provides structure for consistency and transparency in reporting. For further information on Nature Portfolio policies, see our Editorial Policies and the Editorial Policy Checklist.

Statistics

For all statistical analyses, confirm that the following items are present in the figure legend, table legend, main text, or Methods section.

n/a	Confirmed	
☐	☒	The exact sample size (*n*) for each experimental group/condition, given as a discrete number and unit of measurement
☐	☒	A statement on whether measurements were taken from distinct samples or whether the same sample was measured repeatedly
☐	☒	The statistical test(s) used AND whether they are one- or two-sided<br>*Only common tests should be described solely by name; describe more complex techniques in the Methods section.*
☒	☐	A description of all covariates tested
☒	☐	A description of any assumptions or corrections, such as tests of normality and adjustment for multiple comparisons
☐	☒	A full description of the statistical parameters including central tendency (e.g. means) or other basic estimates (e.g. regression coefficient) AND variation (e.g. standard deviation) or associated estimates of uncertainty (e.g. confidence intervals)
☐	☒	For null hypothesis testing, the test statistic (e.g. *F*, *t*, *r*) with confidence intervals, effect sizes, degrees of freedom and *P* value noted<br>*Give P values as exact values whenever suitable.*
☒	☐	For Bayesian analysis, information on the choice of priors and Markov chain Monte Carlo settings
☒	☐	For hierarchical and complex designs, identification of the appropriate level for tests and full reporting of outcomes
☐	☒	Estimates of effect sizes (e.g. Cohen's *d*, Pearson's *r*), indicating how they were calculated

Our web collection on statistics for biologists contains articles on many of the points above.

Software and code

Policy information about availability of computer code

Data collection	No software was used for data collection
Data analysis	GraphPad Prism (version 9.0, for Windows, GraphPad Software, San Diego California) was used for statistical analysis, Microsoft excel was used for data analysis.

For manuscripts utilizing custom algorithms or software that are central to the research but not yet described in published literature, software must be made available to editors and reviewers. We strongly encourage code deposition in a community repository (e.g. GitHub). See the Nature Portfolio guidelines for submitting code & software for further information.

Data

Policy information about availability of data

All manuscripts must include a data availability statement. This statement should provide the following information, where applicable:
- Accession codes, unique identifiers, or web links for publicly available datasets
- A description of any restrictions on data availability
- For clinical datasets or third party data, please ensure that the statement adheres to our policy

All data generated or analyzed during this study are included in the publication or supplementary files.

Human research participants

Policy information about studies involving human research participants and Sex and Gender in Research.

Reporting on sex and gender	N/A
Population characteristics	N/A
Recruitment	N/A
Ethics oversight	N/A

Note that full information on the approval of the study protocol must also be provided in the manuscript.

Field-specific reporting

Please select the one below that is the best fit for your research. If you are not sure, read the appropriate sections before making your selection.

☒ Life sciences ☐ Behavioural & social sciences ☐ Ecological, evolutionary & environmental sciences

For a reference copy of the document with all sections, see nature.com/documents/nr-reporting-summary-flat.pdf

Life sciences study design

All studies must disclose on these points even when the disclosure is negative.

Sample size	No sample size calculator was used to determine sample sizes. For in vivo survival studies, a minimum of 5 mice per group, per experiment were used.
Data exclusions	No data was excluded from the analyses.
Replication	All attempts at reproducibility were successful. Biological and technical replicates were completed as a standard part of the study design and those annotations are contained within the figure legends.
Randomization	Mice were randomized two weeks prior to tumor implantation.
Blinding	Animal studies were not blinded during data collection.

Reporting for specific materials, systems and methods

We require information from authors about some types of materials, experimental systems and methods used in many studies. Here, indicate whether each material, system or method listed is relevant to your study. If you are not sure if a list item applies to your research, read the appropriate section before selecting a response.

Materials & experimental systems

n/a	Involved in the study	
☐	☒	Antibodies
☐	☒	Eukaryotic cell lines
☒	☐	Palaeontology and archaeology
☐	☒	Animals and other organisms
☒	☐	Clinical data
☒	☐	Dual use research of concern

Methods

n/a	Involved in the study	
☒	☐	ChIP-seq
☒	☐	Flow cytometry
☒	☐	MRI-based neuroimaging

Antibodies

Antibodies used

Antibody Target Source Catalog # Dilution Application
B7-H3 (AF1397) R&D Systems AF1397 1:2000 WB
B7-H3 (MIL33B) In house N/A 1:1000-5000 WB
GAPDH (G9545) Sigma Aldrich G9545-100UL 1:10,000 WB
B-Actin (13E5) Cell Signaling Technology 4970 1:10,000 WB
HA-tag (C29F4) Cell Signaling Technology 3724 1:500 WB

GFP-tag (D5.1) Cell Signaling Technology 2956 1:500 WB
Goat-anti-mouse BioRad L005680 1:10,000 WB
Goat anti-Rabbit BioRad 10000045946 1:10,000 WB
Rabbit anti-Goat BioRad L006330A 1:10,000 WB
B7-H3 (AF1397) R&D Systems AF1397 1:400 IF, IHC
B7-H3 (MIL33B) In house N/A 1:200-500 IF, IHC
B7-H3 (D9M2L) Cell Signaling Technology 14058 1:100 IHC

Validation

Primary antibodies that were commercially available were used for the indicated and validated applications. Antibody developed in house was validated by direct binding, and ELISAs to the protein target as well as other family members. WT and KO target cells were also used to identify the contribution of back end binding to the signal.

Eukaryotic cell lines

Policy information about cell lines and Sex and Gender in Research

Cell line source(s)

Cell lines were obtained from MD Anderson Cell Line Repositories, or ATCC. Study generated lines were developed form verified stocks and validated following manipulation.

Authentication

STR validation was performed for all cell lines and confirmed periodically throughout the study timeframe.

Mycoplasma contamination

Cells were tested and found to be negative for mycoplasma contamination multiple times per year throughout the duration of the study.

Commonly misidentified lines
(See ICLAC register)

N/A

Animals and other research organisms

Policy information about studies involving animals; ARRIVE guidelines recommended for reporting animal research, and Sex and Gender in Research

Laboratory animals

Six week-old athymic nude mice (strain nu/nu, Charles River) were anesthetized (1.5% isoflurane inhalation) during tumor cell implantation. 5×106 HeLa cervical cancer cells were injected subcutaneously in the rear flank in 100 µL serum-free media. Tumors were measured (L x W) twice weekly using a caliper and mouse weight was monitored throughout the study. Tumor volume was calculated by the modified ellipsoidal formula: V = ½ (Length x Width2). Six week-old athymic nude mice (strain nu/nu, Charles River) were injected intra-peritoneally with 1.0 x106 SKOv3-ip-FLuc cells/mouse in an orthotopic fashion.

Wild animals

No wild animals were used in this study.

Reporting on sex

Female mice were used as study focused on gynecological cancers.

Field-collected samples

N/A

Ethics oversight

The IACUC board is an internal independent review board at University of Texas MD Anderson Cancer Center, and the Iaucuc protocol number if 00001179 for these experiments.

Accreditation and Assurance Documents

The University of Texas MD Anderson's animal care and use program has been accredited since 1969 by the Association for Assessment and Accreditation of Laboratory Animal Care (AAALAC). The U.S. Department of Agriculture (USDA) inspects the institution for adequate veterinary care. The Office of Laboratory Animal Welfare (OLAW) reviewed and approved the institution's Animal Welfare Assurance submitted in compliance with the Public Health Service (PHS) policy on Humane Care and Use of Laboratory Animals. Related accreditation and assurance documents are provided below.

USDA Inspection Reports available upon request to Office of Research Oversight & Integrity (OROI). Email IACUC.

USDA Inspection Report Certificate
No. #74-R-0065
Last USDA Inspection Date: March 29, 2022

AAALAC Accreditation
No. #000183
Last AAALAC site visit March 2022

PHS Animal Welfare Assurance Statement
A3343-01
Expires April 30, 2026

Note that full information on the approval of the study protocol must also be provided in the manuscript.
